# Effects of Child and Maternal Histo-Blood Group Antigen Status on Symptomatic and Asymptomatic Enteric Infections in Early Childhood

**DOI:** 10.1093/infdis/jiz072

**Published:** 2019-02-15

**Authors:** Josh M Colston, Ruthly Francois, Nora Pisanic, Pablo Peñataro Yori, Benjamin J J McCormick, Maribel Paredes Olortegui, Md Amran Gazi, Erling Svensen, Mondar Maruf Moin Ahmed, Esto Mduma, Jie Liu, Eric R Houpt, Robert Klapheke, Julia W Schwarz, Robert L Atmar, Robert E Black, Margaret N Kosek

**Affiliations:** 1 Department of International Health, Johns Hopkins Bloomberg School of Public Health, Baltimore, Maryland; 2 Department of International Health, Johns Hopkins School of Public Health, Baltimore, Maryland; 3 Department of Environmental Health and Engineering, Johns Hopkins School of Public Health, Baltimore, Maryland; 4 Division of Infectious Diseases and International Health, University of Virginia, Charlottesville; 5 Consultant, Fogarty International Center, Bethesda, Maryland; 6 Asociación Benéfica Prisma, Iquitos, Peru; 7 International Centre for Diarrhoeal Disease Research, Bangladesh; 8 Haukeland University Hospital, Norway; 9 Haydom Global Health Institute, Haydom, Tanzania; 10 University of San Diego, California; 11 Mount Sinai School of Medicine, New York; 12 Baylor College of Medicine, Houston, Texas

**Keywords:** randomized controlled clinical trial, controlled human infection model, *Escherichia coli* infections, diarrhea, prevention and control, fimbriae proteins, colonization factor antigens, antibodies, bacterial, milk proteins, immunology, immunization, passive, bacterial vaccines

## Abstract

**Background:**

Histo-blood group antigens (HBGAs) such as fucosyltransferase (FUT)2 and 3 may act as innate host factors that differentially influence susceptibility of individuals and their offspring to pediatric enteric infections.

**Methods:**

In 3 community-based birth cohorts, FUT2 and FUT3 statuses were ascertained for mother-child dyads. Quantitative polymerase chain reaction panels tested 3663 diarrheal and 18 148 asymptomatic stool samples for 29 enteropathogens. Cumulative diarrhea and infection incidence were compared by child (n = 520) and mothers’ (n = 519) HBGA status and hazard ratios (HRs) derived for all-cause diarrhea and specific enteropathogens.

**Results:**

Children of secretor (FUT2 positive) mothers had a 38% increased adjusted risk of all-cause diarrhea (HR = 1.38; 95% confidence interval (CI), 1.15–1.66) and significantly reduced time to first diarrheal episode. Child FUT2 and FUT3 positivity reduced the risk for all-cause diarrhea by 29% (HR = 0.81; 95% CI, 0.71–0.93) and 27% (HR = 0.83; 95% CI, 0.74–0.92), respectively. Strong associations between HBGAs and pathogen-specific infection and diarrhea were observed, particularly for noroviruses, rotaviruses, enterotoxigenic *Escherichia coli*, and *Campylobacter jejuni*/*coli*.

**Conclusions:**

Histo-blood group antigens affect incidence of all-cause diarrhea and enteric infections at magnitudes comparable to many common disease control interventions. Studies measuring impacts of interventions on childhood enteric disease should account for both child and mothers’ HBGA status.

## Teaser

The histo-blood group antigens of children and their mothers are determinants of diarrhea in early childhood. This study estimates the innate immunity provided by HBGA for principal etiologies of diarrhea and demonstrates altered risk for bacterial and viral enteropathogens.

Diarrheal disease has been a major cause of illness and premature death throughout history [1]. Despite improvements in water and sanitation, oral rehydration therapy, and the rotavirus vaccine, diarrhea illness still claims 500 000 lives of children a year with only modest declines in morbidity [2, 3]. The variability observed in the effectiveness of preventive interventions [4, 5] and in pathogen-specific diarrhea incidence between populations suggests that inherited host factors may differentially influence susceptibility to certain enteric infections [6–10], and a cluster of fucosyltransferase (FUT) genes—primarily the FUT2 and FUT3 genes—have emerged as candidates [11]. Furthermore, population-based genetic analyses reveal widespread selection and nonneutral evolution of the histo-blood group antigen (HBGA) genes similar only to genes involved in antigen recognition [12].

**Figure 1.**
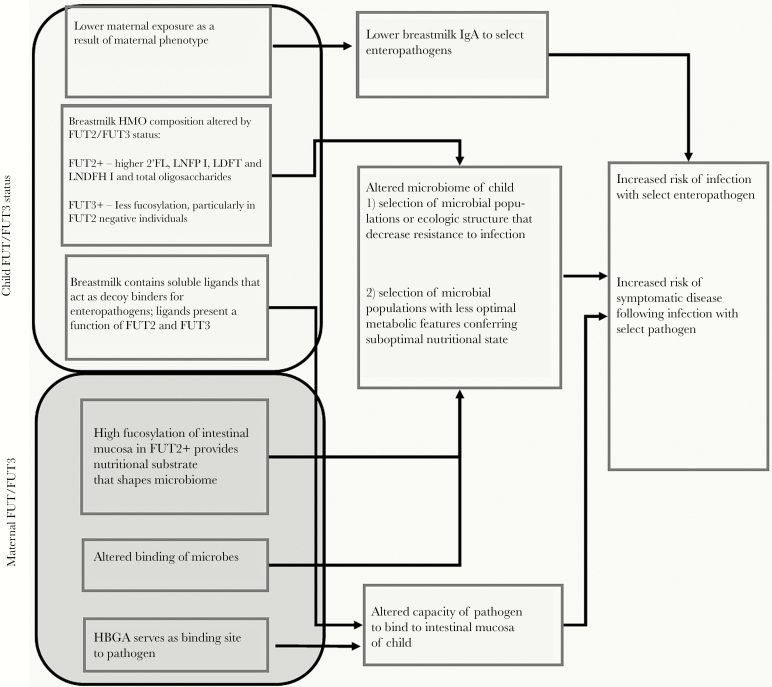
Hypothesized pathways through which maternal and child fucosyltransferase (FUT)2 and FUT3 expression alter susceptibility to enteric infection. 2’-FL, 2’-fucosyllactose; LDFT, lactodifucotetraose; LNDFH I, lacto-*N*-difucohexaose I; LNFP I, lacto-*N*-fucopentaose I [38, 43–45].

**Figure 2.**
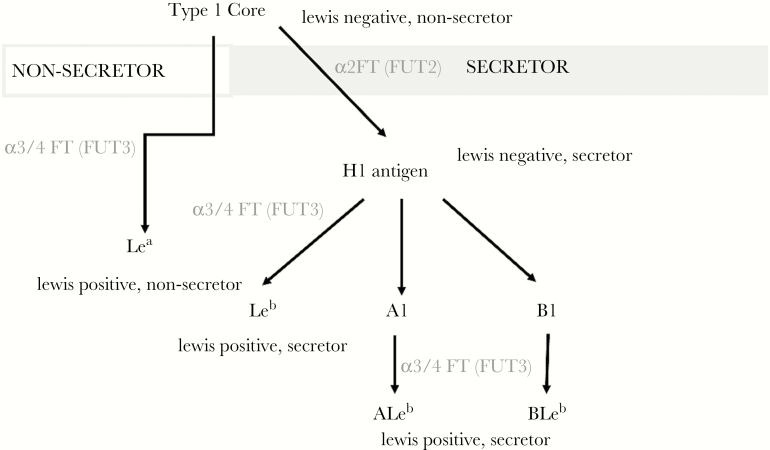
Pathway of histo-blood group antigen synthesis with assignment of phenotype (Lewis and secretor status) in accordance with the presence of a functional phenotype or allele for the FUT2 gene (encoding α (1,2) fucosyltransferase) and FUT3 gene (encoding enzyme with α(1,3) and α(1,4) fucosyltransferase activities).

There are multiple hypothesized pathways by which both an infant’s FUT2 and FUT3 genes and those of their nursing mother may alter susceptibility to enteric infection (Figure 1). The FUT2 gene encodes a 2-α-l-fucosyltransferase, an enzyme required for the surface expression of ABO HBGAs on mucosal membranes and in secreted body fluids (Figure 2). HBGA expression on the intestinal epithelia they may serve as host receptors to which enteric viruses and bacteria attach to establish infection [12–16], and individuals expressing at least 1 functional copy of the FUT2 gene are called secretors. Individuals that are homozygous for nonfunctional FUT2 alleles (nonsecretors) do not produce these HBGA on the intestinal epithelia, which confers a degree of innate protection against enteric infection with some noroviruses [8, 13, 15, 17, 18], P[4] and P[8]-type rotavirus [14, 19], enterotoxigenic *Escherichia coli* (ETEC) [20], and *Campylobacter* [21] and altering the efficacy of the oral rotavirus vaccine [22]. Fucosyltransferase 3, along with other glycosyltransferases, also modifies HBGAs in mucosal secretions and epithelial cell surfaces [16]. In addition, FUT2 and FUT3 phenotype combinations in nursing mothers influence the distribution and concentration of human milk oligosaccharides (HMOs) expressed in breastmilk. These changes in breastmilk composition can durably alter the child’s microbiome [23, 24], which in turn may alter resistance to enteric infections. Furthermore, these glycans have been shown to bind to certain enteropathogens, such as rotavirus, thereby serving as decoy receptors that prevent infections from becoming patent or symptomatic [11, 21, 25].

The analysis reported here assesses these hypotheses by quantifying and modeling associations between infant and maternal secretor (FUT2) status and Lewis (FUT3) type and episodes of infection with the 5 enteric viruses and 5 enteric bacteria most strongly associated with diarrhea: adenovirus, astrovirus, norovirus, rotavirus, sapovirus, and *Campylobacter*, enteroaggregative *E coli* (EAEC), enteropathogenic *E coli* ([EPEC] typical and atypical), heat-labile enterotoxigenic *E coli* (LT-ETEC) and heat-stable ETEC (ST-ETEC), and *Shigella* [10]. The study was done in 3 community-based birth cohorts and used high-resolution diagnostic panels.

## METHODS

### Study Population

The Etiology Risk Factors and Interactions of Enteric Infections and Malnutrition and the Consequences for Child Health and Development (MAL-ED) project investigates risk factors for enteric infection, diarrheal disease, undernutrition, and related outcomes [26]. Birth cohorts were recruited from communities in 8 low- and middle-income countries, 3 of which sites were included in this substudy to capture differences in distributions of genetic polymorphisms in South Asian, African, and South American contexts: Dhaka, Bangladesh (BGD), Loreto, Peru (PEL), and Haydom, Tanzania (TZH), described in previous publications [27–29]. Subjects were enrolled and monitored continuously over their first 2 years of life from November 2009 to March 2014. Written informed consent was obtained from the caregiver of all participating children.

### Ethical Standards

Ethical approval for MAL-ED was given by the Johns Hopkins Institutional Review Board as well as from the respective partner institutions for each site including the following: (BGD) the Institutional Review Board for Health Science Research of the University of Virginia and the Ethical Review Committee of ICDDR,B; (PEL) the Institutional Review Board of the Johns Hopkins School of Public Health, the Ethics Committee of Asociacion Benefica PRISMA, and the Regional Health Department of Loreto; (TZH) the Institutional Review Board for Health Sciences Research of the University of Virginia and the Medical Research Coordinating Committee of the National Institute for Medical Research, and the Ministry of Health and Social Welfare of Tanzania. Written consent was obtained from all participants.

### Outcome Variables

Subjects’ stool samples were collected monthly (surveillance samples) and upon reporting of diarrheal episodes by the caregiver. Subjects and their caregivers were visited twice weekly from enrollment (within 17 days of birth) until 2 years of age. Enteropathogen infection status was ascertained by several methods. Samples were assessed prospectively (1) for adenovirus, astrovirus, rotavirus, and *Campylobacter* by enzyme-linked immunosorbent assay, for *Shigella* by culture, (2) for *E coli* pathotypes by culture with polymerase chain reaction (PCR) confirmation, and (3) for norovirus genogroups I and II by reverse-transcription PCR, as described elsewhere [26, 30]. Samples from children who completed 24 months of follow-up were retrospectively tested for 29 enteropathogens (including sapovirus) and, where possible, strains (throughout this article, we use the generic term “strain” to collectively refer to genotypes of rotavirus, genogroups of norovirus, and species of *Campylobacter*) using probe-based quantitative PCR (qPCR) assays on custom-developed TaqMan Array Cards (Thermo Fisher) [31]. Results from qPCR (75.7% of total observations) were preferentially used in these analyses where available; otherwise, results from the prospective assessment were substituted. To ensure that a single infection episode was not counted multiple times, *Campylobacter*- and norovirus-positive samples were excluded if they were collected within 30 days of a previous sample that was positive for the same pathogen strain without being separated by an intermediate negative sample. For all other pathogens, a 14-day period was used. Because the TaqMan Array uses the same gene target for *Shigella* and enteroinvasive *E coli* (EIEC), these 2 related pathogens were also collapsed into 1 outcome (*Shigella*).

### Covariates

The main exposures of interest in this analysis were secretor (FUT2) status and Lewis (FUT3) type ascertained for study subjects and their mothers from saliva samples using a phenotyping assay [32] in PEL and BGD and sequencing of the FUT2 and FUT3 genes in TZH where specimen collection methods did not allow for the assay to be performed (as detailed in Appendix 1). Phenotypes were classified according to the criteria in Supplemental Table 1a and b. Subjects whose phenotype could not be ascertained were genotyped according to methods described by Reeck et al [32]. In a secondary analysis, blood type was treated as an exposure and categorized as a binary variable comparing group O with groups A, B, and AB (the reference category). In addition, the following a priori-selected covariates were used in the analysis: (1) age: the infants’ age in continuous months at the time of sample collection was treated as the analysis-time variable in all survival analyses; (2) sample type: multivariate analyses were stratified by surveillance and diarrheal stool samples to separately model effects on symptomatic and asymptomatic episodes; (3) study site: a categorical variable indicating whether the subject was enrolled in the BGD (reference category), PEL, or TZH cohort included to adjust for between-site variation in both prevalence of gene expressions and pathogen transmission rates; (4) breastfeeding status: a categorical variable indicating whether, on the day before stool sample collection, subjects were breastfed exclusively, partially (reference category), predominantly, or not at all. (A small number of missing values for breastfeeding status were imputed based on the most common status for that 6-month age group and site.)

### Statistical Methods

Using survival analysis, which allowed for multiple failures per subject, Kaplan-Meier estimates were plotted comparing cumulative incidence of diarrhea of any etiology (“all-cause diarrhea”) and individual pathogens and strains between FUT phenotype-positive and negative subjects and subjects’ mother across the first 2 years of life. Hazard ratios (HRs) from univariate, unstratified Cox proportional hazard models were also reported. Multivariate Cox regression models were then fitted for all-cause diarrhea and individual pathogens adjusting mothers’ and infants’ FUT2 and FUT3 status for each other and for covariates and stratifying by sample type (diarrheal or surveillance stools). Separately, stratified Cox models were fitted comparing O with A/B/AB blood groups for those sites where such information was available (BGD and PEL) among secretors adjusting for covariates but not FUT statuses. For 3 pathogen strains with reported binding sites for H Type I antigens, hypothesized to be present at greatest concentration in FUT2^+^/FUT3^−^ individuals—P[4]/P[8] rotavirus, CFA/I^+^ ETEC, and *Campylobacter jejuni*/*coli*—and for all-cause diarrhea, separate Cox models were fitted comparing HRs of Lewis normal to null type among secretor children (see Figure 2). Analyses were carried out using Stata 13.1 [33].

## RESULTS

The FUT2 status was ascertained for 534 of the 827 children enrolled at the 3 sites and for 521 of their mothers, whereas 520 children and 519 mothers had FUT3 status ascertained (Table 1). No nonsecretor children and only 1 nonsecretor mother was identified in PEL; however, Lewis null prevalence was highest there of the 3 sites with 21.2% of children and 25.0% of mothers with ascertained FUT3 status. Among subjects for whom FUT2 status was ascertained, prevalence of nonsecretor children was similar in BGD and TZH (22.5% and 19.1%, respectively) but differed substantially among mothers (14.5% in BGD, 22.4% in TZH).

**Table 1.** Distribution of Child and Maternal FUT Status Combinations in 3 Birth Cohorts

Country				Child’s Status				Mother’s Status			
				FUT2^+^	FUT2^−^	FUT3^+^	FUT3^−^	FUT2^+^	FUT2^−^	FUT3^+^	FUT3^−^
Bangladesh	Child’s Status	FUT3^+^	Number	120	38						
			Percentage	(65.2)	(20.7)						
		FUT3^−^	Number	23	3						
			Percentage	(12.5)	(1.6)						
	Mother’s Status	FUT2^+^	Number	119	28	123	22				
			Percentage	(69.2)	(16.3)	(72.4)	(12.9)				
		FUT2^−^	Number	13	12	24	1				
			Percentage	(7.6)	(7.0)	(14.1)	(0.6)				
		FUT3^+^	Number	119	37	137	18	131	25		
			Percentage	(69.2)	(21.5)	(80.6)	(10.6)	(76.2)	(14.5)		
		FUT3^−^	Number	13	3	10	5	16	0		
			Percentage	(7.6)	(1.7)	(5.9)	(2.9)	(9.3)	(0.0)		
	Total		Number	145	42	158	26	147	25	156	16
			Percentage	(77.5)	(22.5)	(85.9)	(14.1)	(85.5)	(14.5)	(90.1)	(9.9)
Peru	Child’s status	FUT3^+^	Number	152	0						
			Percentage	(78.8)	(0.0)						
		FUT3^−^	Number	41	0						
			Percentage	(21.2)	(0.0)						
	Mother’s Status	FUT2^+^	Number	189	0	148	39				
			Percentage	(99.5)	(0.0)	(78.7)	(20.7)				
		FUT2^−^	Number	1	0	1	0				
			Percentage	(0.5)	(0.0)	(0.5)	(0.0)				
		FUT3^+^	Number	142	0	124	18	143	1		
			Percentage	(75.1)	(0.0)	(66.3)	(9.6)	(74.5)	(0.5)		
		FUT3^−^	Number	47	0	24	21	48	0		
			Percentage	(24.9)	(0.0)	(12.8)	(11.2)	(25.0)	(0.0)		
	Total		Number	195	0	152	41	192	1	144	48
			Percentage	(100.0)	(0.0)	(78.8)	(21.2)	(99.5)	(0.5)	(75.0)	(25.0)
Tanzania	Child’s Status	FUT3^+^	Number	101	22						
			Percentage	(71.6)	(15.6)						
		FUT3^−^	Number	13	5						
			Percentage	(9.2)	(3.5)						
	Mother’s Status	FUT2^+^	Number	98	19	95	14				
			Percentage	(64.9)	(12.6)	(66.9)	(9.9)				
		FUT2^−^	Number	24	10	29	4				
			Percentage	(15.9)	(6.6)	(20.4)	(2.8)				
		FUT3^+^	Number	114	27	118	16	115	31		
			Percentage	(76.0)	(18.0)	(83.7)	(11.3)	(74.2)	(20.0)		
		FUT3^−^	Number	7	2	5	2	5	4		
			Percentage	(4.7)	(1.3)	(3.5)	(1.4)	(3.2)	(2.6)		
	Total		Number	123	29	125	18	121	35	146	9
			Percentage	(80.1)	(19.9)	(87.4)	(12.6)	(77.6)	(22.4)	(94.2)	(5.8)
Total				463	71	435	85	460	61	446	73
Not ascertained				293		307		306		308	

Abbreviations: FUT, fucosyltransferase.

**Figure 3.**
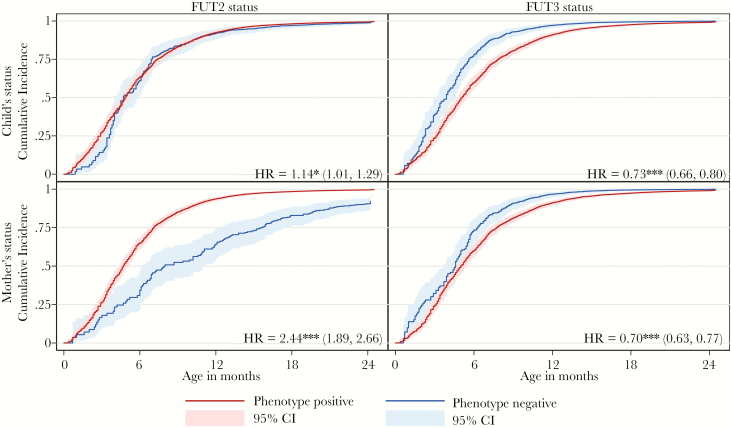
Kaplan-Meier plot of cumulative incidence of diarrhea of any etiology by age for phenotype positive and negative subjects by fucosyltransferase (FUT) gene and for child’s and mother’s status with hazard ratios (HRs) comparing phenotype positive to negative status from univariate Cox regression models (with 95% confidence interval [CI] [***, *P* < .001, **, *P* = .001–.01, and *, *P* = .01–.05] and significance level). Maternal FUT2 status consistently had the largest association with the risk of all-cause diarrhea, with risk ratios greatest between 3 and 12 months of age but significant until 24 months of age.

Enteroaggregative *E coli* was the most prevalent pathogen in both diarrheal and surveillance stools overall and within each of the sites followed by *Campylobacter* (Table 2). Of the 10 pathogens analyzed, typical EPEC had the lowest prevalence in diarrheal stools, whereas among asymptomatic samples, rotavirus prevalence was lowest. GII norovirus was more prevalent than GI in all sites, and both stool types and atypical GII strains were more common than GII.4. The most commonly isolated rotavirus G-types in diarrheal stools overall were G9 and G12; however, this was mainly due to their high prevalence in the BGD site, where they had isolation rates of 4.2% and 4.0%, respectively. In TZH, G1 rotavirus was by far the most commonly isolated G-strain in both stool types. In PEL, G2 was most common in diarrheal, and G1 was most common in surveillance samples. Among rotavirus P-types, P[8]—the Rotarix vaccine target—was the most commonly isolated in all sites and both sample types, although diarrheal P[4] isolates were much more prevalent in BGD than in the other 2 sites (none were recorded in TZH). Supplemental Figure 1 shows incidence of each outcome before 6 months of age by FUT status and in participants for whom status was not ascertained. There was no evidence of infection incidence differing by ascertainment status. Figure 3 shows Kaplan-Meier plots of the cumulative incidence of all-cause diarrhea by age comparing child and mother FUT2 and FUT3 phenotypes along with HRs from univariate, unstratified Cox models. The FUT2-positive (secretor) infants, and those with secretor mothers, had a statistically significantly increased risk of diarrhea and reduced time to first diarrheal episode. This was especially marked for maternal FUT2 status. At 9 months of age, a cumulative incidence of 85.2% (95% confidence interval [CI], 83.2%–87.1%) was observed in children of secretor mothers compared with 52% (95% CI, 42.3%–63.2%) in children of nonsecretors (log-rank equality test, *P* ≤ .0001). Overall, children of secretors had 2.24 times (95% CI, 1.89–2.66) the risk of diarrhea episodes than those of nonsecretors. A reduced risk of all-cause diarrhea was seen in nonsecretor compared with secretor children before approximately 5 months of age, but not subsequently. Both maternal and child’s Lewis status had large and highly statistically significant protective effects against diarrhea. Lewis-positive infants had a 27% lower risk of all-cause diarrhea (HR = 0.73; 95% CI, 0.66–0.80) compared with Lewis null infants, and the cumulative incidences for the 2 groups at 9 months of age were 80.1% (95% CI, 78.3%–83.1%) and 92.9% (95% CI, 90.0%–95.2%), respectively. For children of Lewis normal and Lewis null mothers, the equivalent statistics were 70% (HR = 0.70; 95% CI, 0.63–0.77), 81.3% (95% CI, 78.9%–83.6%), and 91.0% (95% CI, 87.0%–93.9%). Equivalent Kaplan-Meier estimates and HRs for each pathogen and strain are in Appendix 2.

**Table 2.** Numbers and Percentages of Stool Samples That Were Positive for Enteropathogens in 3 MAL-ED Study Sites and Overall

Enteropathogen	BGD		PEL		TZH		Total	
	n	(%)	n	(%)	n	(%)	n	(%)
Adenovirus 40/41								
Asymptomatic infections	927	(16.2)	1140	(16.6)	462	(8.3)	2529	(13.9)
Diarrheal episodes	651	(40.9)	458	(23.3)	14	(13.0)	1123	(30.7)
Astrovirus								
Asymptomatic infections	926	(16.1)	827	(12.0)	334	(6.0)	2087	(11.5)
Diarrheal episodes	420	(26.4)	449	(22.8)	8	(7.4)	877	(23.9)
Norovirus—Either Genogroup								
Asymptomatic infections	871	(15.2)	945	(13.8)	806	(14.5)	2622	(14.4)
Diarrheal episodes	356	(22.4)	607	(30.9)	23	(21.3)	986	(26.9)
GI Norovirus								
Asymptomatic infections	302	(5.3)	281	(4.1)	216	(3.9)	799	(4.4)
Diarrheal episodes	122	(7.7)	181	(9.2)	5	(4.6)	308	(8.4)
GII.4 Norovirus								
Asymptomatic infections	70	(1.2)	125	(1.8)	129	(2.3)	324	(1.8)
Diarrheal episodes	35	(2.2)	64	(3.3)	2	(1.9)	101	(2.8)
Other GII Norovirus								
Asymptomatic infections	564	(9.8)	519	(7.6)	465	(8.4)	1548	(8.5)
Diarrheal episodes	169	(10.6)	219	(11.1)	12	(11.1)	400	(10.9)
Rotavirus—Any Genotype								
Asymptomatic infections	270	(4.7)	180	(2.6)	246	(4.4)	696	(3.8)
Diarrheal episodes	351	(22.1)	105	(5.3)	17	(15.7)	473	(12.9)
G1 Rotavirus								
Asymptomatic infections	29	(0.5)	27	(0.4)	70	(1.3)	126	(0.7)
Diarrheal episodes	15	(0.9)	4	(0.2)	6	(5.6)	25	(0.7)
G2 Rotavirus								
Asymptomatic infections	5	(0.1)	23	(0.3)	7	(0.1)	35	(0.2)
Diarrheal episodes	11	(0.7)	25	(1.3)	0	(0.0)	36	(1.0)
G3 Rotavirus								
Asymptomatic infections	0	(0.0)	20	(0.3)	2	(0.0)	22	(0.1)
Diarrheal episodes	0	(0.0)	20	(1.0)	0	(0.0)	20	(0.5)
G4 Rotavirus								
Asymptomatic infections	0	(0.0)	0	(0.0)	2	(0.0)	2	(0.0)
Diarrheal episodes	0	(0.0)	0	(0.0)	0	(0.0)	0	(0.0)
G8 Rotavirus								
Asymptomatic infections	0	(0.0)	1	(0.0)	5	(0.1)	6	(0.0)
Diarrheal episodes	0	(0.0)	0	(0.0)	0	(0.0)	0	(0.0)
G9 Rotavirus								
Asymptomatic infections	44	(0.8)	7	(0.1)	1	(0.0)	52	(0.3)
Diarrheal episodes	67	(4.2)	10	(0.5)	0	(0.0)	77	(2.1)
G12 Rotavirus								
Asymptomatic infections	30	(0.5)	2	(0.0)	16	(0.3)	48	(0.3)
Diarrheal episodes	64	(4.0)	4	(0.2)	0	(0.0)	68	(1.9)
P[4] Rotavirus								
Asymptomatic infections	21	(0.4)	26	(0.4)	5	(0.1)	52	(0.3)
Diarrheal episodes	47	(3.0)	31	(1.6)	0	(0.0)	78	(2.1)
P[6] Rotavirus								
Asymptomatic infections	13	(0.2)	9	(0.1)	8	(0.1)	30	(0.2)
Diarrheal episodes	15	(0.9)	10	(0.5)	0	(0.0)	25	(0.7)
P[8] Rotavirus								
Asymptomatic infections	45	(0.8)	30	(0.4)	64	(1.2)	139	(0.8)
Diarrheal episodes	92	(5.8)	22	(1.1)	5	(4.6)	119	(3.2)
Sapovirus								
Asymptomatic infections	798	(13.9)	737	(10.7)	470	(8.5)	2005	(11.0)
Diarrheal episodes	358	(22.5)	350	(17.8)	16	(14.8)	724	(19.8)
*Campylobacter* spp								
Asymptomatic infections	1763	(30.7)	1287	(18.7)	2130	(38.4)	5180	(28.5)
Diarrheal episodes	597	(37.5)	552	(28.1)	41	(38.0)	1190	(32.5)
EAEC								
Asymptomatic infections	3326	(58.0)	3361	(48.9)	3546	(64.0)	10233	(56.4)
Diarrheal episodes	837	(52.6)	1005	(51.1)	71	(65.7)	1913	(52.2)
Atypical EPEC								
Asymptomatic infections	1178	(20.5)	1419	(20.7)	1404	(25.3)	4001	(22.0)
Diarrheal episodes	301	(18.9)	404	(20.6)	30	(27.8)	735	(20.1)
Typical EPEC								
Asymptomatic infections	966	(16.8)	664	(9.7)	848	(15.3)	2478	(13.7)
Diarrheal episodes	285	(17.9)	235	(12.0)	19	(17.6)	539	(14.7)
LT-ETEC								
Asymptomatic infections	803	(14.0)	1009	(14.7)	1336	(24.1)	3148	(17.3)
Diarrheal episodes	235	(14.8)	384	(19.5)	29	(26.9)	648	(17.7)
ST-ETEC								
Asymptomatic infections	1477	(25.7)	595	(8.7)	1275	(23.0)	3347	(18.4)
Diarrheal episodes	589	(37.0)	233	(11.9)	29	(26.9)	851	(23.2)
*Shigella* spp/EIEC								
Asymptomatic infections	587	(10.2)	596	(8.7)	797	(14.4)	1980	(10.9)
Diarrheal episodes	318	(20.0)	243	(12.4)	15	(13.9)	576	(15.7)
Total stool samples								
Surveillance collections	5736	(31.6)	6871	(37.9)	5541	(30.5)	18148	(100.0)
Diarrheal collections	1590	(43.4)	1965	(53.6)	108	(2.9)	3663	(100.0)

Abbreviations: BGD, Bangladesh; *E coli*, *Escherichia coli*; EAEC, enteroaggregative *E coli*; EIEC, enteroinvasive *E coli*; EPEC, enteropathogenic *E coli*; LT-ETEC, heat-labile enterotoxigenic *E coli*; MAL-ED, Malnutrition and the Consequences for Child Health and Development; PEL, Peru; ST-ETEC, heat-stable ETEC; TZH, Tanzania.

**Figure 4.**
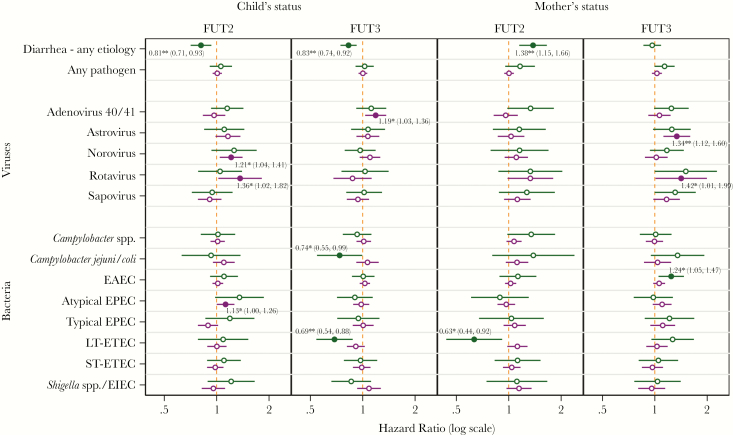
Hazard ratios for diarrhea and for infection with common enteric pathogens comparing phenotype positive to negative status from multivariate Cox models that adjusted for mothers’ fucosyltransferase (FUT)2 and FUT3 status (for child estimates) and child’s FUT2 and FUT3 (for maternal estimates) and covariates stratified by diarrheal (green) and surveillance (purple) stool samples. Statistically significant estimates (*P* ≤ .05) are represented by solid markers and labeled with the hazard ratio (with 95% confidence interval; ***, *P* < .001, **, *P* = .001–.01, and *, *P* = .01–.05) and significance level.

After adjustment for the child’s Lewis type and maternal secretor status and Lewis type, study site, and breastfeeding status, the child’s secretor status was associated with a 19% risk reduction for all-cause diarrhea (HR = 0.81; 95% CI, 0.71–0.93) during the first 2 years of life (Figure 4), a significant change from the univariate model findings. The similarly adjusted association of child’s FUT3 status demonstrated a 27% reduction in all-cause diarrhea risk (HR = 0.83; 95% CI, 0.74–0.92) in Lewis-positive compared with Lewis null children, consistent with the univariate model. Children who were secretors had a statistically significant 21% increased risk of asymptomatic norovirus (HR = 1.21; 95% CI, 1.04–1.41) but not norovirus diarrhea. However, secretor children had a 36% increased risk of asymptomatic rotavirus illness from all genotypes (HR = 1.36; 95% CI, 1.02–1.82) and a 13% increased risk of asymptomatic atypical EPEC (HR = 1.13; 95% CI, 1.00–1.26) relative to nonsecretors. A reduction in *C jejuni/coli* diarrhea risk was seen in Lewis normal infants (HR = 0.74; 95% CI, 0.55–0.99), but not in asymptomatic infection, or in infection from combined *Campylobacter* species. Lewis normal infants also experienced a decreased risk (HR = 0.69; 95% CI, 0.54–0.88) of LT-ETEC diarrhea, but not of asymptomatic LT-ETEC infection, and an increased risk of asymptomatic adenovirus (HR = 1.19; 95% CI, 1.03–1.36), but not of adenovirus diarrhea.

Maternal secretor-positive status conferred an excess all-cause diarrhea risk of 38% (HR = 1.38; 95% CI, 1.15–1.66) to children after adjusting for other phenotypes and covariates. The only pathogen-specific association with maternal secretor-positive status was seen for LT-ETEC with children of secretors showing a 27% reduced risk of LT-ETEC diarrhea than those of nonsecretors (HR = 0.63; 95% CI, 0.44–0.92). Maternal FUT3 was no longer associated with all-cause diarrhea risk in the multivariate model but conferred a 34% increased risk of astrovirus infection (HR = 1.34; 95% CI, 1.12–1.60), but not astrovirus diarrhea. Children of Lewis normal mothers showed a 42% increased risk of rotavirus diarrhea (HR = 1.42; 95% CI, 1.01–1.99), but no difference in asymptomatic infections, and a 24% increase in EAEC diarrhea (HR = 1.24; 95% CI, 1.05–1.47). No associations were observed between any maternal or child HBGA status and risk of infection or diarrhea with sapovirus, typical EPEC, ST-ETEC, or *Shigella*.

**Figure 5.**
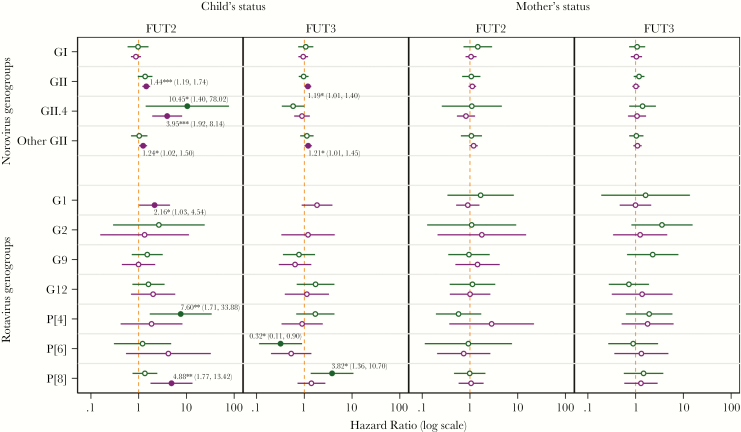
Hazard ratios (HRs) for infection with common virus strains comparing phenotype positive to negative status from multivariate Cox models that adjusted for child and mothers’ fucosyltransferase (FUT)2 and FUT3 status and covariates stratified by diarrheal (green) and surveillance (purple) stool samples. Statistically significant estimates (*P* ≤ .05) are represented by solid markers and labeled with the HR (with 95% confidence interval; ***, *P* < .001, **, *P* = .001–.01, and *, *P* = .01–.05) and significance level. Diarrhea was not noted in children who were Lewis positive with G1 rotavirus infections, thus a HR was undefined. (Just 1 case each of G1 and G2 rotavirus diarrhea was observed in nonsecretor infants, and no cases of G1 rotavirus diarrhea were observed in Lewis null infants, so it was not possible to calculate HRs for those associations.)

When identical multivariate models were fitted to evaluate associations between maternal and child HBGA status and risk of strain-specific rotavirus and norovirus infections, no associations were observed for maternal statuses, but numerous strong associations were observed for the child’s status (Figure 5A). The most marked increase in risk among secretor compared with nonsecretor children was for GII.4 norovirus diarrhea (HR = 10.45; 95% CI, 1.40–78.02). Asymptomatic norovirus GII.4 infection was also 3.95 times more common in secretor than nonsecretor children (HR = 3.95; 95% CI, 1.92–8.14). Child secretor status conferred a 24% increased risk (HR = 1.24; 95% CI, 1.02–1.50) of asymptomatic infection of less common GII noroviruses, but not for diarrheal episodes. There were no associations between GI noroviruses and any HBGA status. Child secretor status conferred almost 8 times the risk of P[4] rotavirus diarrhea (HR = 7.60; 95% CI, 1.71–33.88) and 5 times the risk of asymptomatic P[8] rotavirus infection (HR = 4.88; 95% CI, 1.77–13.42). A child’s Lewis-positive status increased the adjusted risk of diarrheal P[8] rotavirus by almost fourfold (HR = 3.82; 95% CI, 1.36–10.70), but not the risk of asymptomatic infection. No diarrhea attributable to rotavirus G1 was seen in Lewis null children, and just 1 such episode was seen in secretor children. Lewis-positive children were at a 68% decreased risk (HR = 0.32; 95% CI, 0.11–0.90) of symptomatic diarrhea from rotavirus P[6], but no difference was observed in the risk of asymptomatic infection. Lewis normal children had a 21% greater risk of infections (HR = 1.21; 95% CI, 1.00–1.45) with atypical GII norovirus, but not diarrhea caused by these infections.

There was also evidence of differential susceptibility of children to ETEC infections and diarrhea due to ETEC based on the specific identity of colonization antigens/fimbrae as a function of the child’s, but not maternal, secretor status (Appendix 5). Secretor children had 1.79 (95% CI, 1.03–3.12) times the risk of ETEC diarrhea associated with CFA/1, 4.91 (95% CI, 1.12–21.49) times the risk of CS5-producing ETEC diarrhea, and 1.88 (95% CI, 1.14–3.08) times the risk of CS6 compared with nonsecretors. No effect was observed of Lewis type or maternal HBGA status on any individual ETEC fimbria.

In the models comparing blood group O subjects to those of other groups (Appendices 3 and 4), no significant effects on individual pathogens were observed except a 24% reduction in adenovirus diarrhea (HR = 0.76; 95% CI, 0.61–0.95) and a 69% reduction in P[8] rotavirus diarrhea (HR = 0.31; 95% CI, 0.14–0.70). However, a 21% increase in all-cause diarrhea risk was observed for blood group O subjects (HR = 1.21; 95% CI, 1.06–1.38). Because multiple enteric pathogens (rotavirus P[4], P[6], P[8] [34, 35], and *C jejuni* [21]) have been described as binding to H-Type I HBGA, which would be maximally expressed in Lewis null secretor children. We compared infection and diarrhea from these pathogens between FUT2^+^/FUT3^−^ and FUT2^+^/FUT3^+^ children (see Figure 2). The HR associated with being a Lewis null secretor for P[8] rotavirus diarrhea was 5.65 (95% CI, 1.77–18.10) compared with Lewis normal secretors, whereas the equivalent for P[4] rotavirus diarrhea was 2.45 (95% CI, 1.04–5.82) demonstrating that although binding has been described to both Le^b^ and Type I antigens [7, 36, 37], the epidemiologic data strongly supports the binding to Type I antigen as being of primary importance in determining innate susceptibility to disease. However, no such effect was observed for either *C jejuni/coli* or other species of *Campylobacter*, another species with purported binding the Type I antigen [21].

## DISCUSSION

In this analysis of a longitudinal study of 827 children on 3 continents, we demonstrate that HBGAs of both the child and the child’s mother were important determinants of all-cause diarrhea, as well as incidence of several important diarrhea-associated enteric pathogens. Although many of these associations are consistent with those described or hypothesized previously, the empirical results of this study yield more precise, population-level effect size estimates than have been available to date, as well as being suggestive of hitherto unidentified associations of HBGAs with less well studied pathogens such as adenovirus, astrovirus, EAEC, and atypical EPEC. The use of a PCR array card with simultaneous detection of a large panel of pathogens and adherence factors is a strength of the current study because it allowed for the relative risk of different microbes and genotypes to be compared with each other in the same populations. The inclusion of 3 populations with relatively large genetic differences is a second strength of the current work.

Although previous studies have examined associations between HBGAs and individual viruses and their strains in vivo [19] and single bacteria species in vitro [21], this is the first study to increase the scope to account for the effect of both secretor and Lewis status in both members of the mother-child dyad on asymptomatic and diarrheal episodes and utilize comprehensive enteric pathogen detection methods across 3 geographically and genetically diverse populations.

An unexpected finding was that maternal secretor status showed a stronger association with a child’s risk of diarrhea than the equivalent status of the child itself. Furthermore, upon adjustment for other HBGA statuses and covariates, this effect remained strong and statistically significant and in the opposing direction to the protective effects of both the child’s own secretor status and their Lewis type. Although the apparent protection conferred by a child’s Lewis normal status against all-cause diarrhea appears to be accounted for by the preventive effect on risk of diarrheal *C jejuni/coli*, LT-ETEC, and P[6] rotavirus, the similar effect of secretor status is not explained by any particular pathogen species, a finding for which we lack a clear explanation. It is possible that the diagnostics we did, although comprehensive by current standard, is not detecting some etiologic agents. However, these findings do suggest that decoy binding to pathogen receptors does not explain the protective effect of breastmilk (which is certainly multifactorial), because children of secretor mothers had more all-cause diarrhea and higher pathogen carriage then those of nonsecretors. It appears more likely that glycans affect host susceptibility to infection principally by altering the microbiome through the manipulation of *Bacteroides* species [38].

This study demonstrates that the same FUT phenotype can significantly affect a single outcome in opposing directions depending on whether the child or mother’s status is considered. This is most striking in the qualitatively different effect of maternal (risk greatest in children of secretor mothers) compared with child’s secretor status (risk decreased among secretor children) on all-cause diarrhea. Furthermore, competing effects of a particular HBGA on different strains of the same pathogen were observed. Lewis normal status in a child conferred an increased risk of G1 and P[8] rotavirus but a decreased risk of diarrheal P[6] rotavirus. Such competing effects are likely evidence of balancing selection maintaining the expression of a given HBGA phenotype within particular populations despite their risk for certain individual infections.

Data from a health facility in Dhaka specializing in treatment of moderate to severe pediatric diarrhea from 2015 to 2017 show that, even with high-quality case management, admitted infants aged 0–5 months have a case fatality rate more than 3 times that of those aged 6–11 months—a risk ratio of 3.16 (95% CI, 2.33–4.30) [39]. This suggests that, although cumulative incidence at 2 years may approach 100% for many of the pathogens analyzed here, any factors that prolong the time to first episode until after the critical first 6 months of life—such as differences in breast milk HMO profile—may have the potential to reduce diarrhea-attributable mortality. In contrast, genetic factors that shorten this time may have exerted selective survival pressures on historical populations that universally lacked access to case management. Several of the effects of HBGAs identified here seem to play this role of delaying illness episodes to beyond this critical window for a large proportion of infants, but the effects of FUT2 are most notable. That secretors are more likely but their nonsecretor offspring less likely to survive infancy favors the persistence of nonfunctioning alleles in the population.

## CONCLUSIONS

The effects observed in this analysis are comparable in magnitude to those of common interventions such as rotavirus vaccine, which has an effectiveness at preventing severe disease of approximately 50% in high child mortality settings [40] and water and sanitation improvements and hygiene promotion [41, 42]. The HBGA antigen testing is simple, affordable, and available for implementation at the level of regional laboratories. Researchers assessing the impacts of such interventions should adjust for the distribution of HBGA in both the children in study populations and their mothers, which may act as effect modifiers impacting on their ability to influence outcomes. More detailed characterization of the pathogen- and strain-specific effects of HBGAs on enteric infection can inform the development of precision public health and improve the success of regionalized and targeted interventions.

## Supplementary Data

Supplementary materials are available at *The Journal of Infectious Diseases* online. Consisting of data provided by the authors to benefit the reader, the posted materials are not copyedited and are the sole responsibility of the authors, so questions or comments should be addressed to the corresponding author.

jiz072_Suppl_Supplementary_Material

## Notes

***Acknowledgments.*** We thank the participants, their families, and the study community for their dedicated time and effort to better the understanding the transmission and more enduring impact of enteric infections in early childhood. We also thank the following: Jan Vinje (Centers for Disease Control and Prevention) for critical input and manuscript review; Dr. Leah Jager for consultation regarding the statistical analysis; Dr. Ben Jann (University of Bern, Switzerland) for guidance in generating the figures; Christine Szymanski for insight and encouragement, particularly regarding Campylobacter infection and disease patency; Chris Damman and Anita Zaidi for input on early iterations of the analysis; and Dick Guerrant for final reflections.

***Financial support.*** This work was funded by the Etiology, Risk Factors, and Interactions of Enteric Infections and Malnutrition and the Consequences for Child Health and Development Project (MAL-ED) is carried out as a collaborative project funded by the Bill & Melinda Gates Foundation (BMGF) (BMGF-47075), the Foundation for the National Institutes of Health, and the National Institutes of Health, Fogarty International Center, whereas additional support was obtained from BMGF for the examination of host innate factors on enteric disease risk and enteropathy (Grants OPP1066146 and OPP1152146; to M. N. K.). Additional funding was obtained from teh Sherrilyn and Ken Fisher Center for Environmental Infectious Diseases, Johns Hopkins School of Medicine (to M. N. K) and the National Center for Research Resources and the National Center for Advancing Translational Sciences, National Institues of health 1UL1TR001079.

***Potential conflicts of interest.*** All authors: No reported conflicts of interest. All authors have submitted the ICMJE Form for Disclosure of Potential Conflicts of Interest.
