# Supplementary material for: Effects of Child and Maternal Histo-Blood Group Antigen Status on Symptomatic and Asymptomatic Enteric Infections in Early Childhood
Source: J Infect Dis. 2019 Feb 15;220(1):151–62. doi: 10.1093/infdis/jiz072 (PMC6548901; doi:10.1093/infdis/jiz072)
Supplement: jiz072_Suppl_Supplementary_Material [file jiz072_suppl_supplementary_material.docx]

## **Supplementary materials**

***Appendix 1 - Lewis and secretor status phenotyping and genotyping***

**Saliva collection:** Saliva was collected using the Oracol saliva collection device (Malvern Medical Development) in Peru and Bangladesh by rubbing the sponge along the gum line for 1 min. Samples were kept on ice or refrigerated until processing. Samples were centrifuged sponge up for 10 min at 1,500g and the saliva transferred to a microcentrifuge tube. Saliva was centrifuged again for 5 min at 10,000g; the supernatant was transferred into a separate vial and DNA was extracted from the remaining cell pellet according to instructions of the QIAamp DNA mini kit (Qiagen). In Tanzania saliva was collected using OraSure swabs. The resulting specimen was not compatible with the phenotype assay. Instead, DNA was extracted according to instructions of the QIAamp DNA mini kit (Qiagen).

**Secretor status, Lewis type and ABO phenotyping** [1]**:** Saliva samples were centrifuged for 5 min at 10,000 rpm; 20 µL of the supernatant were boiled for 5 min in a water bath, then placed on ice. Samples were then diluted 1:500 in PBS and vortexed for 5 sec. 100 µL of each diluted sample was added per well before incubating the 96-well plates at room temperature for 4 hours. Within the last hour, 100 µL of 10% non-fat dry milk was added into each well. The plates were blocked overnight at 2-8°C in a humidity chamber.

The plates were washed three times with 230 µL of 0.05% PBS-Tween-20. 100 µL/well of 1:100 Anti-Le^a^ Gamma-clone (ImmucorGamma #4861), 1:100 BG-6 (SIG-3315), 1:200 Anti-A Gamma-clone (ImmucorGamma #0410203), or 1:100 Anti-B Gamma-clone (ImmucorGamma #0413203) was added to the appropriate wells. After a 1-hr incubation at 37°C in a humidity chamber, the plates were washed six times. 100 µL/well of 1:5,000 anti-mouse IgG-peroxidase (Sigma #A2304) or 1:5,000 UEA-1 (lectin from Ulex europaeus)-peroxidase (Sigma #L8146) were added to the corresponding wells. The plates were incubated for 1 hr at 37oC in a humidity chamber then washed six times. Antigens were detected using 100 µL/well of TMB Microwell Peroxidase Substrate System (KPL #507600) with a 10-min incubation at 22-25°C in the dark. The reaction was stopped using 1 M phosphoric acid (100 µL/well), and the optical density read at λ=450nm.

**Lewis and secretor status genotyping:** Secretor (FUT2) and Lewis (FUT3) genotype were determined for individuals with undetermined phenotype. The FUT2 gene was amplified using FUT2_fw 5’-ACA CAC CCA CAC TAT GCC TGC AC-3’ and FUT2_rev 5’-ACT TGC AGC CCA ACG CAT CTT-3’ [2] and FUT3 using FUT3_Amp_fw 5’-GAA ACA GGA ATA ATA GCA GCT CCT C-3’ and FUT3_Amp_rev 5’-TAG CAG GCA AGT CTT CTG GA-3’ [3]. FUT2 and FUT3 were amplified in a 50 µL reaction by PCR using the following conditions: 1 x high GC buffer, 200 nM forward and reverse primers, 10 µM dNTPs, 1.25 units of OneTaq polymerase (New England Biolabs) and 5 µL DNA template. Thermal cycling conditions were 94°C for 30 sec, 40 cycles of 94°C for 15 sec, 58°C (FUT2) / 53°C (FUT3) for 15 sec, and 68°C for 80 sec, then 68°C for 5 min. A 1,264 bp and 1,357 bp fragment were amplified for FUT2 and FUT3, respectively.

Amplicons were sequenced using the primers noted above on an Applied Biosystems 3720xl DNA Analyzer (The Synthesis and Sequencing Facility, Johns Hopkins University School of Medicine, Baltimore MD). Resulting sequences were analyzed using Sequencher 5.4.6 (Gene Codes) and revised manually for heterozygous positions.

| Supplemental table 1a: FUT2 genotype to phenotype interpretation | | | |
| --- | --- | --- | --- |
| Allele^[[1]](#footnote-1)^ | **Secretor Phenotype** | **n** | **Assumptions** |
| se^171,216,(315),428,739,960^ | non-secretor | 6 | se^428^ is non-secretor |
| se^171,216,(315),428,739^ | non-secretor | 1 | se^428^ is non-secretor |
| se^171,216,(382),428,739,960^ | non-secretor | 1 | se^428^ is non-secretor |
| se^171,216,428,(543),739,960^ | non-secretor | 3 | se^428^ is non-secretor |
| se^171,216,428,739,960^ | non-secretor | 52 | se^428^ is non-secretor |
| se^171,216,428,739^ | non-secretor | 1 | se^428^ is non-secretor |
| Se^(40),(113),(171),(216),(428),(739),(960)^ | Secretor | 4 |  |
| Se^(40),(113),(171),(216),(428),(739),(960),(1046)^ | Secretor | 2 |  |
| Se^(40),(171),(216),(315),(428),(739),(960)^ | Secretor | 1 |  |
| Se^(40), (171),(216),(357),(428),(739),(960)^ | Secretor | 1 |  |
| Se^(40), (171),(216),(428),(543),(739),(951),(960)^ | Secretor | 1 |  |
| Se^(40), (171),(216),(428),(739),(951),(960)^ | Secretor | 2 |  |
| Se^(40), (171),(216),(428),(739), (960)^ | Secretor | 20 |  |
| Se^(40),(171),(216),(428),(739),(960),(1046)^ | Secretor | 5 |  |
| Se^171,216,(428),(481),(739),(960)^ | Secretor | 2 |  |
| Se^(171),216,(428),(481),(739),(960)^ | Secretor | 1 |  |
| Se^(171),(216),(315),(357),(428),(739),(960)^ | Secretor | 1 |  |
| Se^(171),(216),(315),(428),(481),(739),(960)^ | Secretor | 2 |  |
| Se^(171),(216),(357),(428),(480),(739),(960)^ | Secretor | 12 |  |
| Se^(171),(216),(357),(428),(480),(739),(960),(1046)^ | Secretor | 2 |  |
| Se^(171),(216),(357),(428),(480),(739),(1046)^ | Secretor | 1 |  |
| Se^(171),(216),(357),(428),(669),(739)^ | Secretor | 1 |  |
| Se^(171),(216),(357),(428),(739),(960)^ | Secretor | 53 |  |
| Se^(171),(216),(357),(428),(739),(960),(1046)^ | Secretor | 10 |  |
| Se^(171),(216),(357),(428),(739)^ | Secretor | 5 |  |
| Se^(171),(216),(428),(481),(739),(960)^ | Secretor | 23 |  |
| Se^(171),(216),(428),(481),(739),(960),(1046)^ | Secretor | 2 |  |
| Se^(171),(216),(428),(739),(960)^ | Secretor | 1 |  |
| Se^(171),(216),(428),(739)^ | Secretor | 1 |  |
| Se^40,113^ | Secretor | 1 |  |
| Se^40,(113),(951)^ | Secretor | 1 |  |
| Se^40,(113)^ | Secretor | 6 |  |
| Se^40,951^ | undetermined | 1 |  |
| Se^40^ | Secretor | 3 |  |
| Se^(40),(113),(357),(480)^ | Secretor | 1 |  |
| Se^(40),(113),(357)^ | Secretor | 6 |  |
| Se^(40),(113),(481)^ | Secretor | 1 |  |
| Se^(40),(357),(480)^ | Secretor | 1 |  |
| Se^(40),(357),(739),(960)^ | Secretor | 1 |  |
| Se^(40),(357),(951)^ | Secretor | 1 |  |
| Se^(40),(357)^ | Secretor | 18 |  |
| Se^(40),(481),(951)^ | Secretor | 2 |  |
| Se^(40),(481)^ | Secretor | 6 |  |
| Se^(171),(216),(357),(481)^ | Secretor | 1 |  |
| Se^(171),(216),(481)^ | Secretor | 1 |  |
| Se^(216),(357),(481)^ | Secretor | 1 |  |
| Se^357,(480)^ | Secretor | 6 |  |
| Se^357,(739),(960),(1046)^ | Secretor | 1 |  |
| Se^357^ | Secretor | 17 |  |
| Se^(357),(480),(481)^ | Secretor | 3 |  |
| Se^(357),(481),(739),(960)^ | Secretor | 1 |  |
| Se^(357),(481)^ | Secretor | 8 |  |
| Se^481^ | Secretor | 6 |  |

| Supplemental table 1b: Lewis genotype to phenotype interpretation | | | |
| --- | --- | --- | --- |
| Allele^[[2]](#footnote-2)^ | **Lewis Phenotype** | **n** | **Assumptions** |
| le^59,508^ | null | 3 |  |
| le^59,(202),(314),(508)^ | null | 1 | likely le^59,508^ & le^59,202,314^ |
| le^59,(508),(1067)^ | likely null | 3 | likely le^59,508^ & le^59,1067^ |
| le^59,(508),(548) ,(1067)^ | likely null | 1 | likely le^59,508^ & le^59,548,1067^ |
| le^59,(876),1067^ | null | 1 | le^1067^ is null |
| le^59,1067^ | null | 2 | le^1067^ is null |
| le^1067*^ | undetermined | 1 | base undetermined |
| le^(59),(202),(314),(508)^ | null | 3 | likely le^59,508^ & le^202,314^ |
| Le^59,(508)^ | positive | 2 | only le^59,508^ null |
| le^(13),(59),(484),(508),(667),(808)^ | null | 1 | likely le^59,508^ & le^13,484,667,808^ |
| Le^(59),(508)^ | positive | 26 | Le^59^ only not null |
| le^(59),(202),(314),(1067)^ | likely null | 7 | likely le^59,1067^ & le^202,314^ |
| Le^(59),(1067)^ | positive | 35 |  |
| Le^(13),(59),(484),(667),(808)^ | positive | 4 |  |
| Le^(59),(202),(314)^ | positive | 1 |  |
| Le^(59),(808)^ | positive | 7 |  |
| Le^(1067)^ | positive | 1 |  |
| Le^(59)^ | positive | 13 |  |
| Le^(13),(59),(667),808^ | positive | 1 |  |
| le^(13),(202),(314),(484),(667),(808)^ | null | 3 | likely le^202,314^ & le^13,484,667,808^ |
| le^202,314^ | null | 1 |  |
| Le^(202),(314)^ | positive | 53 |  |
| Le^(508)^ | positive | 1 |  |
| le^13,484,667,(808)^ | null | 1 |  |
| Le^(13),(484),(667),(808)^ | positive | 9 |  |
| Le^(13),(484),(808)^ | positive | 1 |  |
| Le^(13),(667),(808)^ | positive | 7 |  |
| Le | positive | 112 |  |

**References:**

1. Reeck A, Kavanagh O, Estes MK, Opekun AR, Gilger MA, Graham DY, et al. Serological correlate of protection against norovirus-induced gastroenteritis. J. Infect. Dis. [Internet]. 2010;202:1212–8. https://academic.oup.com/jid/article-lookup/doi/10.1086/656364: Accessed: 2018 Aug 17

2. Ferrer-Admetlla A, Sikora M, Laayouni H, Esteve A, Roubinet F, Blancher A, et al. A Natural History of FUT2 Polymorphism in Humans. Mol. Biol. Evol. [Internet]. 2009;26:1993–2003. http://www.ncbi.nlm.nih.gov/pubmed/19487333: Accessed: 2018 Jun 11

3. Matzhold EM, Helmberg W, Wagner T, Drexler C, Ulrich S, Winkler A, et al. Identification of 14 new alleles at the fucosyltransferase 1, 2, and 3 loci in Styrian blood donors, Austria. Transfusion [Internet]. 2009;49:2097–108. http://doi.wiley.com/10.1111/j.1537-2995.2009.02293.x: Accessed: 2018 Aug 17

***Appendix 3 – Distribution of blood types for secretor infants at the two sites for which such information was available***

|  | Bangladesh | | Peru | | Total | |
| --- | --- | --- | --- | --- | --- | --- |
| Blood group | **n** | **(%)** | **n** | **(%)** | **n** | **(%)** |
| A | 50 | (36.5) | 11 | (6.4) | 61 | (19.7) |
| B | 28 | (20.4) | 3 | (1.7) | 31 | (10.0) |
| AB | 29 | (21.2) | 0 | (0.0) | 29 | (9.35) |
| O | 30 | (21.9) | 159 | (91.9) | 189 | (61.0) |
| Total | 137 | (100.0) | 173 | (100.0) | 310 | (100.0) |

***Appendix 4 - Hazard ratios for diarrhea and for infection with common enteric pathogens comparing O with A/B/AB blood groups for those sites where such information was available (Bangladesh and Peru) among secretors adjusting for covariates but not FUT statuses stratified by diarrheal (green) and surveillance (purple) stool samples. Statistically significant estimates (p ≤ 0.05) are represented by solid markers and labeled with the hazard ratio (with 95% CI) and significance level.^^[[3]](#footnote-3)^^***


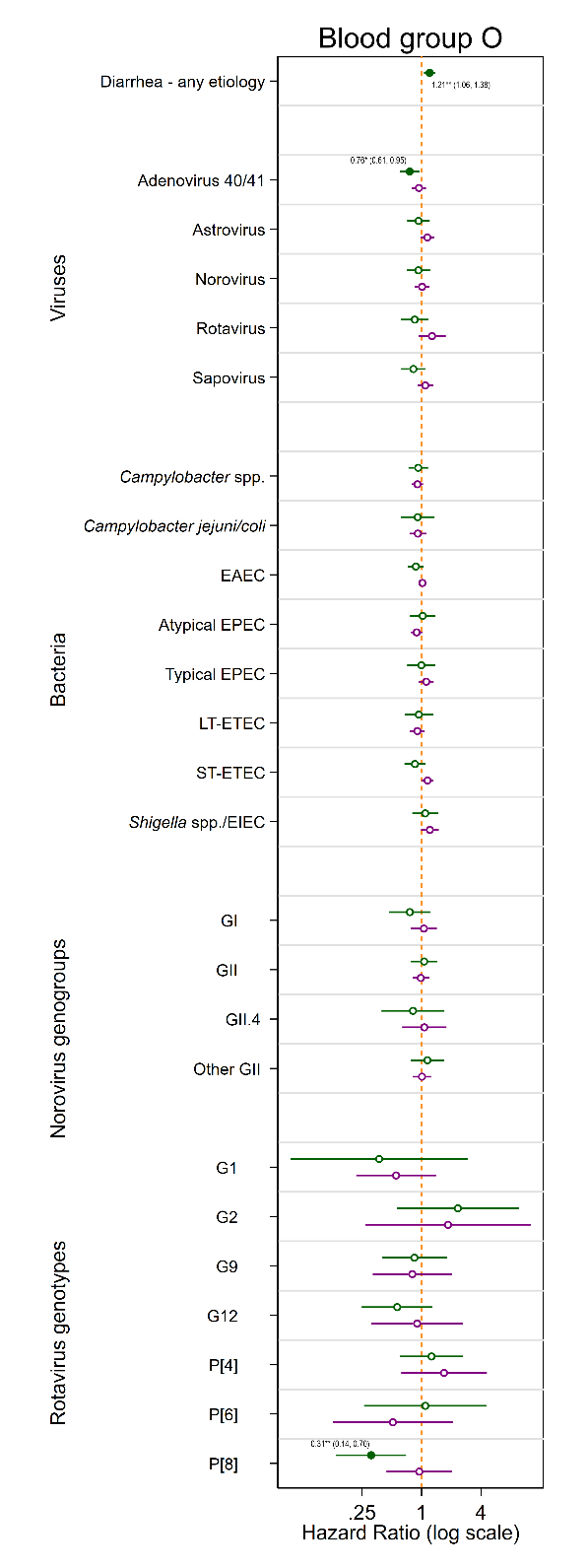


***Appendix 5 - Hazard ratios for diarrhea and for infection with ETEC CFA/I and related CF fimbriae comparing phenotype positive to negative status from multivariate Cox models that adjusted for maternal FUT2 and FUT3 status (for child estimates) and child’s FUT2 and FUT3 (for maternal estimates) and covariates stratified by diarrheal (green) and surveillance (purple) stool samples. Statistically significant estimates (p ≤ 0.05) are represented by solid markers and labeled with the hazard ratio (with 95% CI) and significance level.^^[[4]](#footnote-4)^^***

**
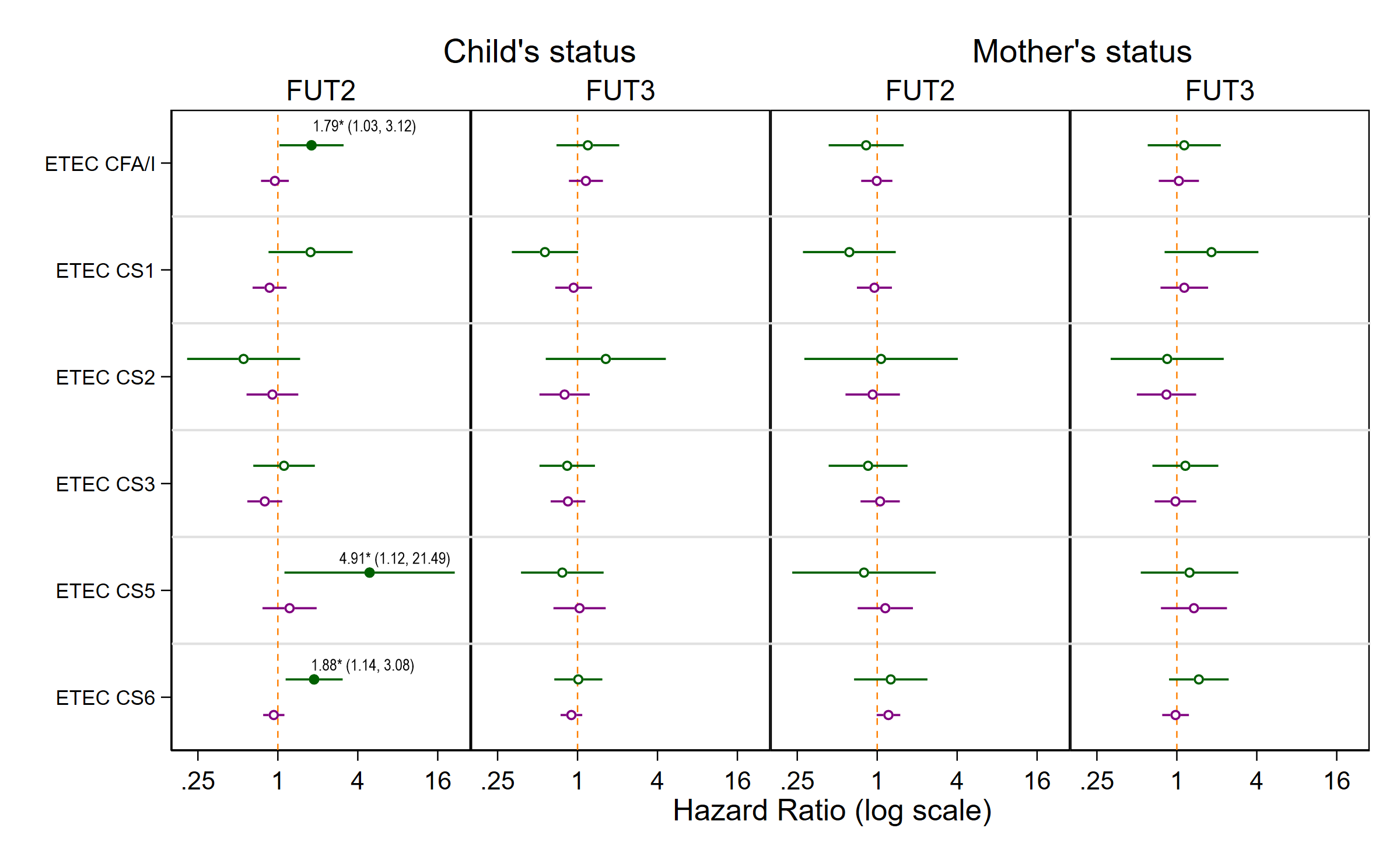
**

***Appendix 6 – Supplemental figure 1: Incidence rates (per person-month) of diarrhea and common enteric pathogens in infants under 6 months in the 3 cohorts by maternal and child’s FUT status, in participants for whom status was not ascertained and overall***

***
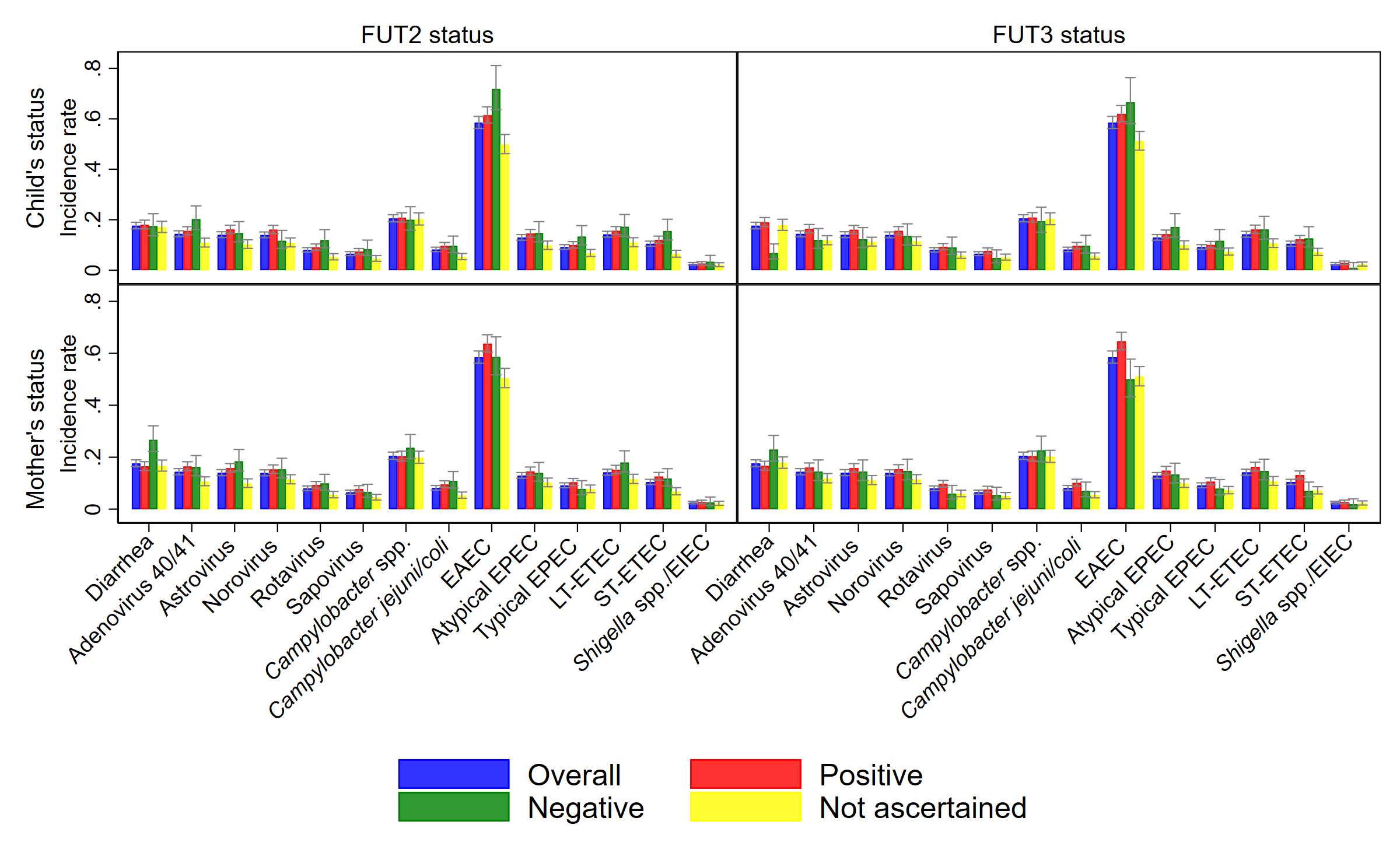
***

***Appendix 7 – Kaplan-Meier plots of cumulative incidence of enteric pathogens by age for phenotype positive and negative subjects by FUT gene and for child's and mother's status with hazard ratios (HRs) from univariate Cox regression models (with 95% CI) and significance level***


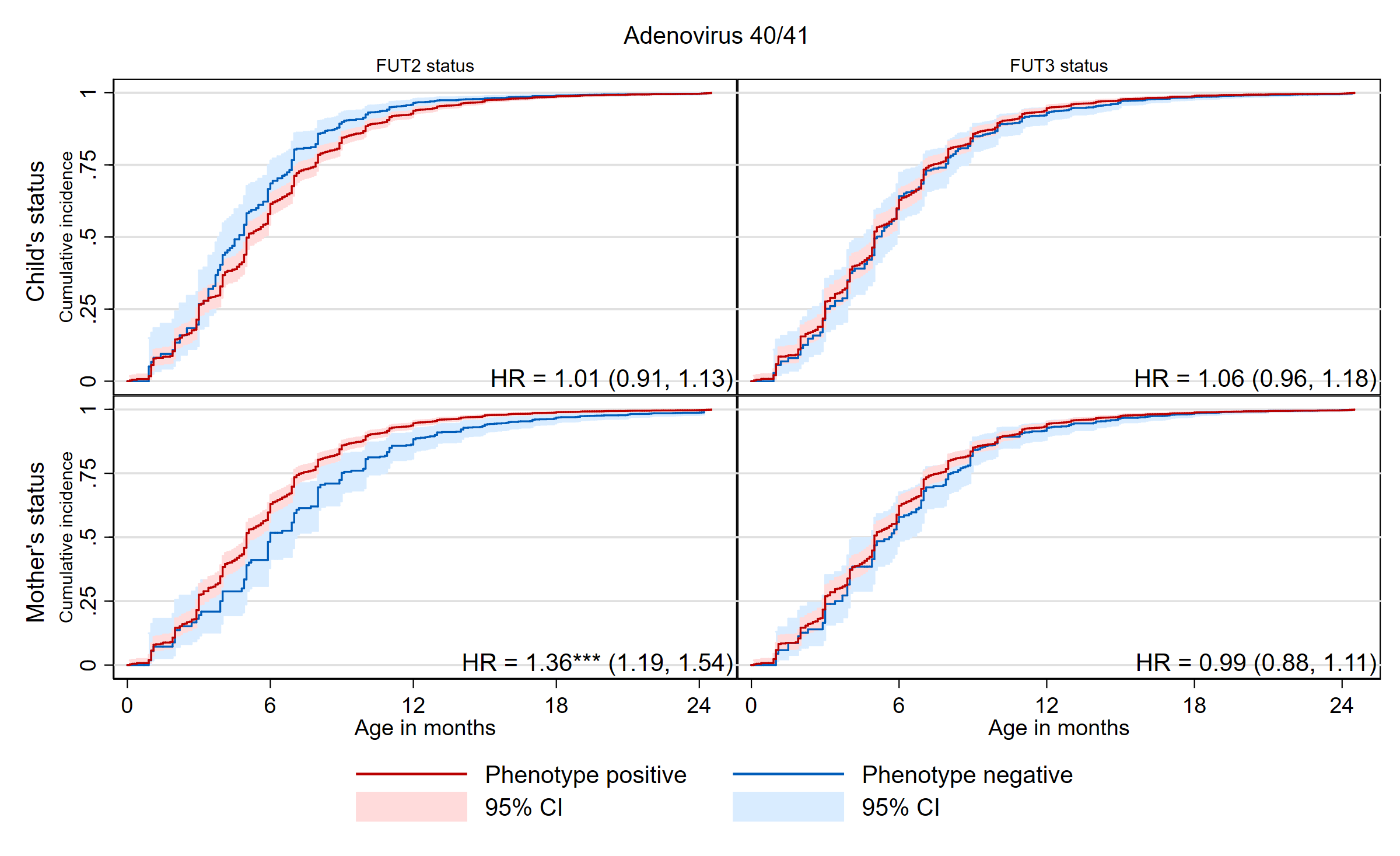


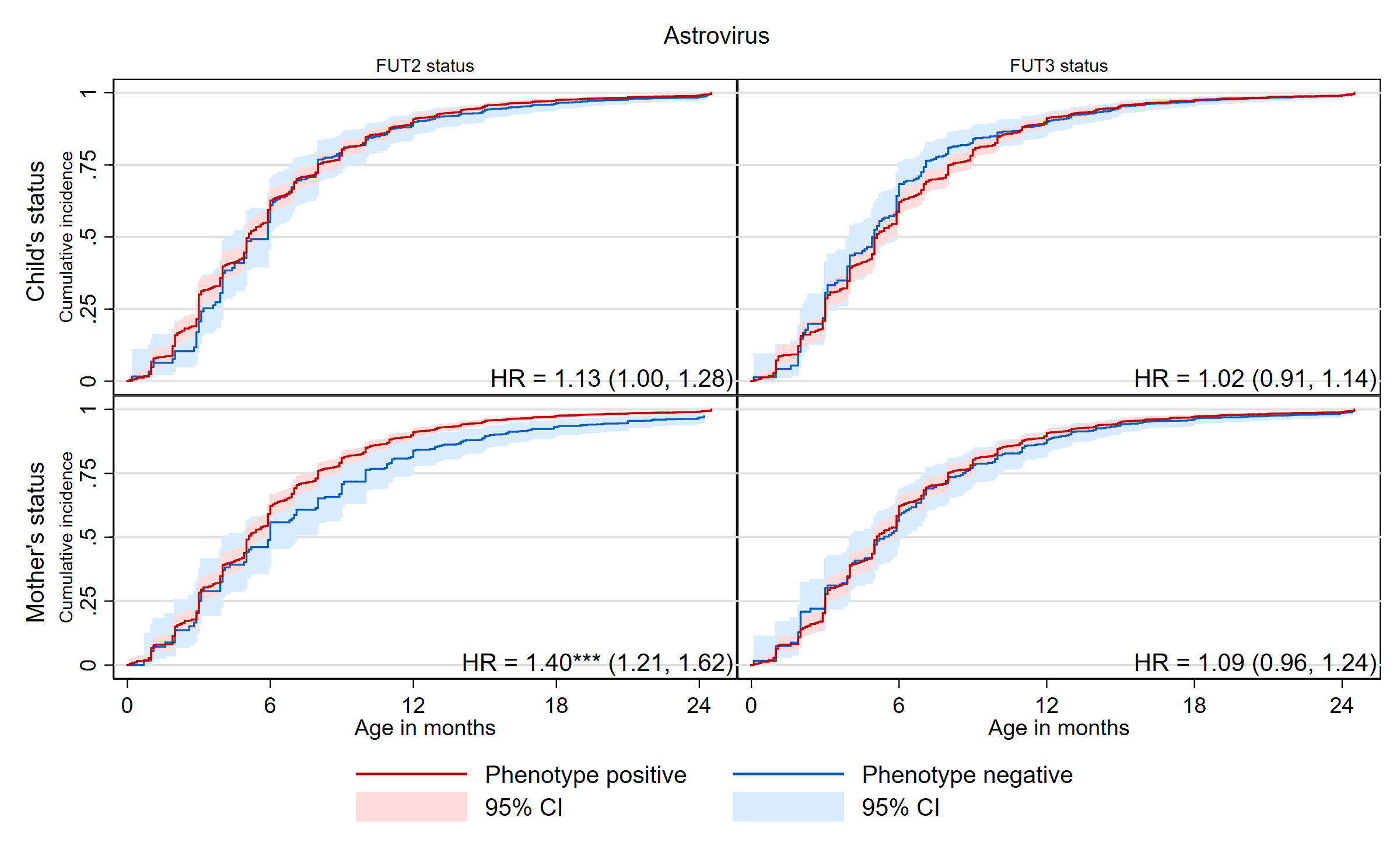


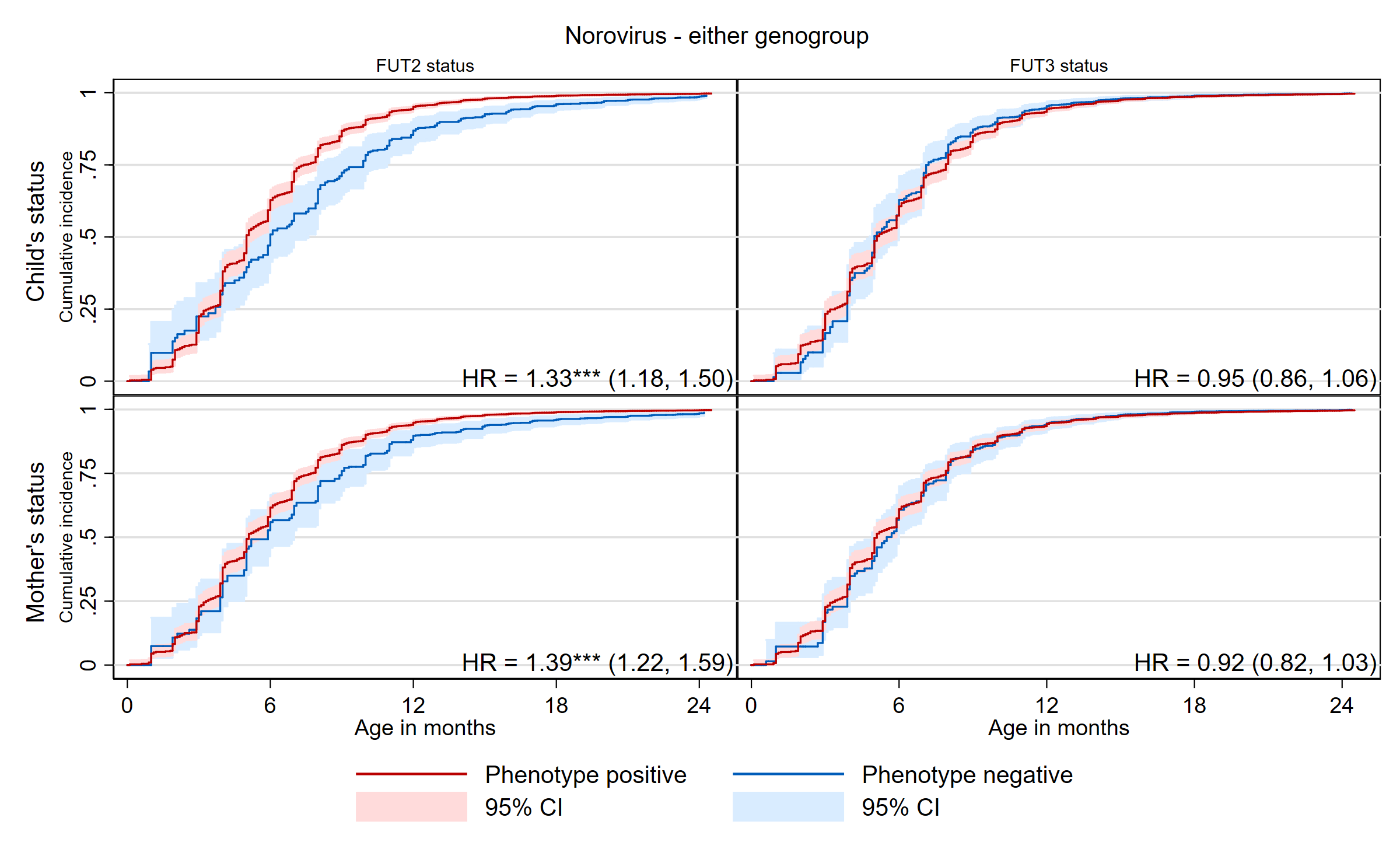


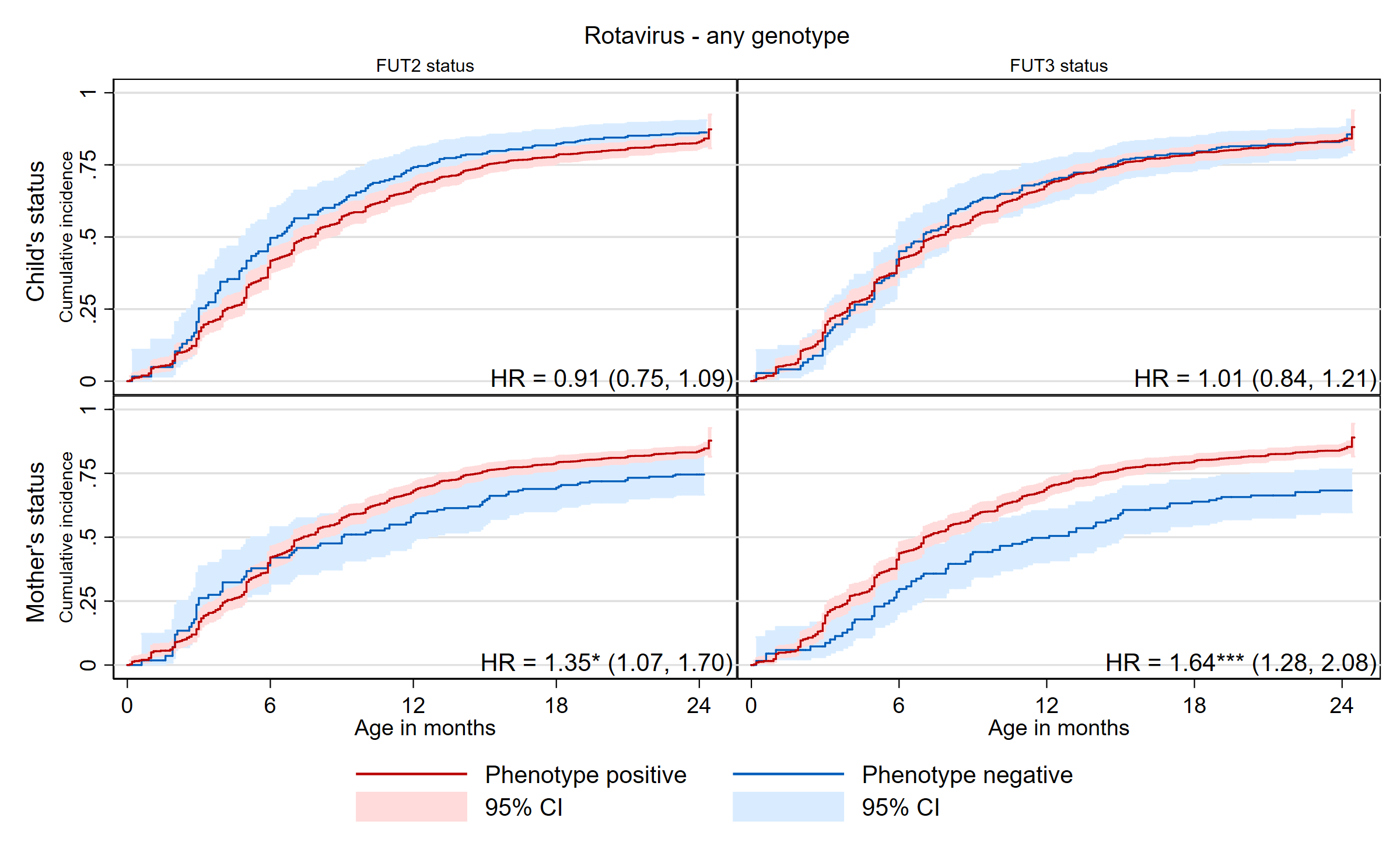


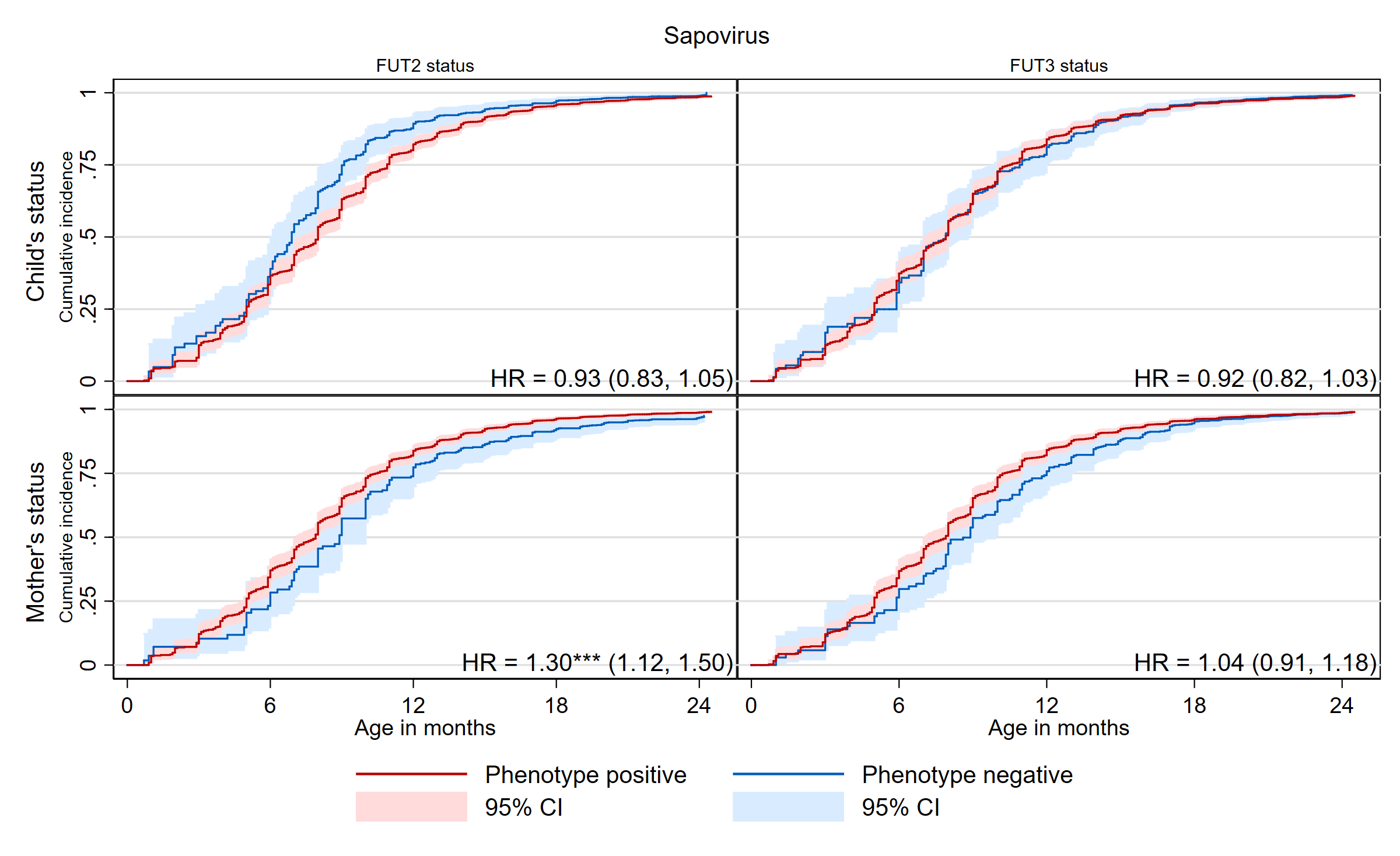


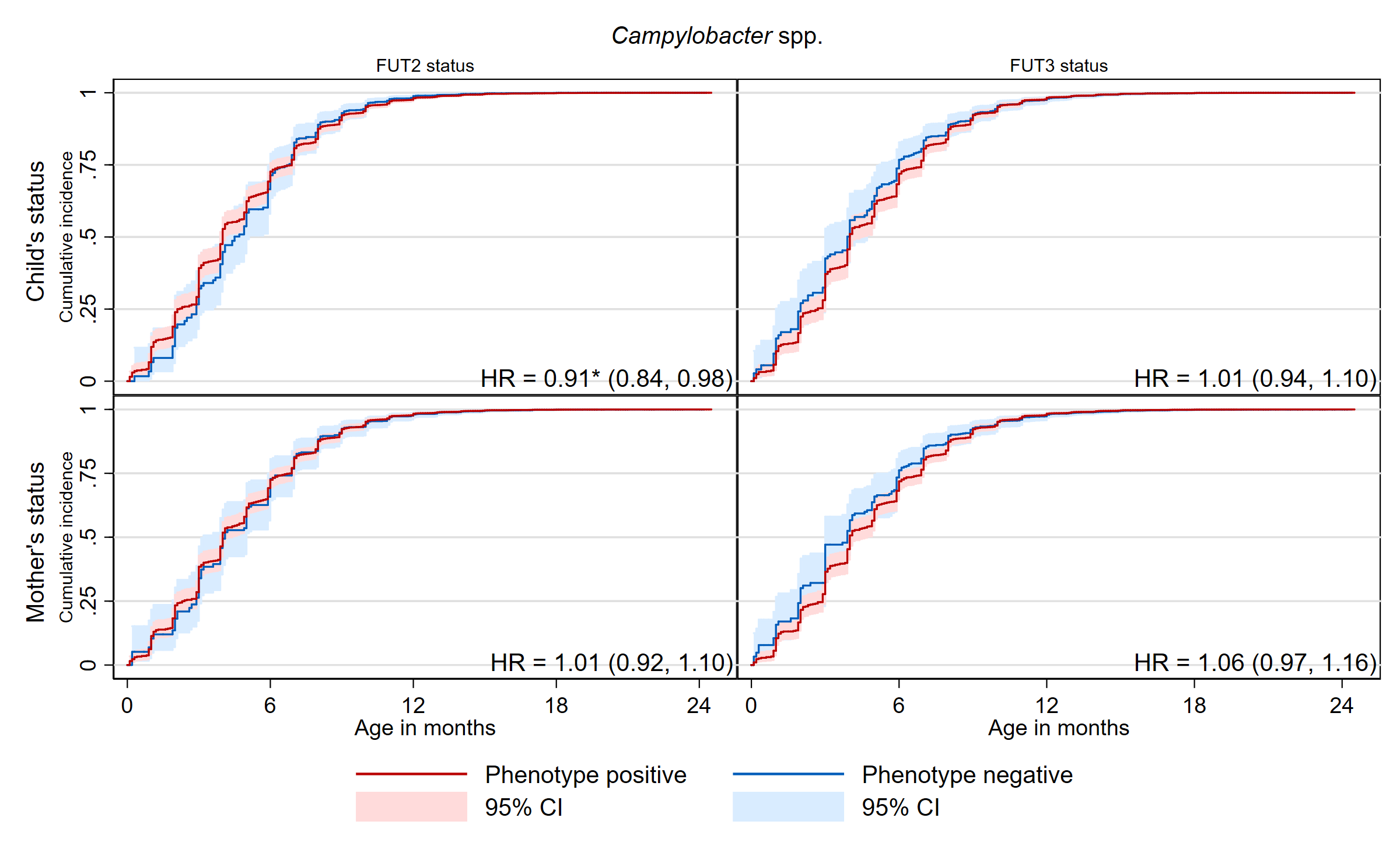


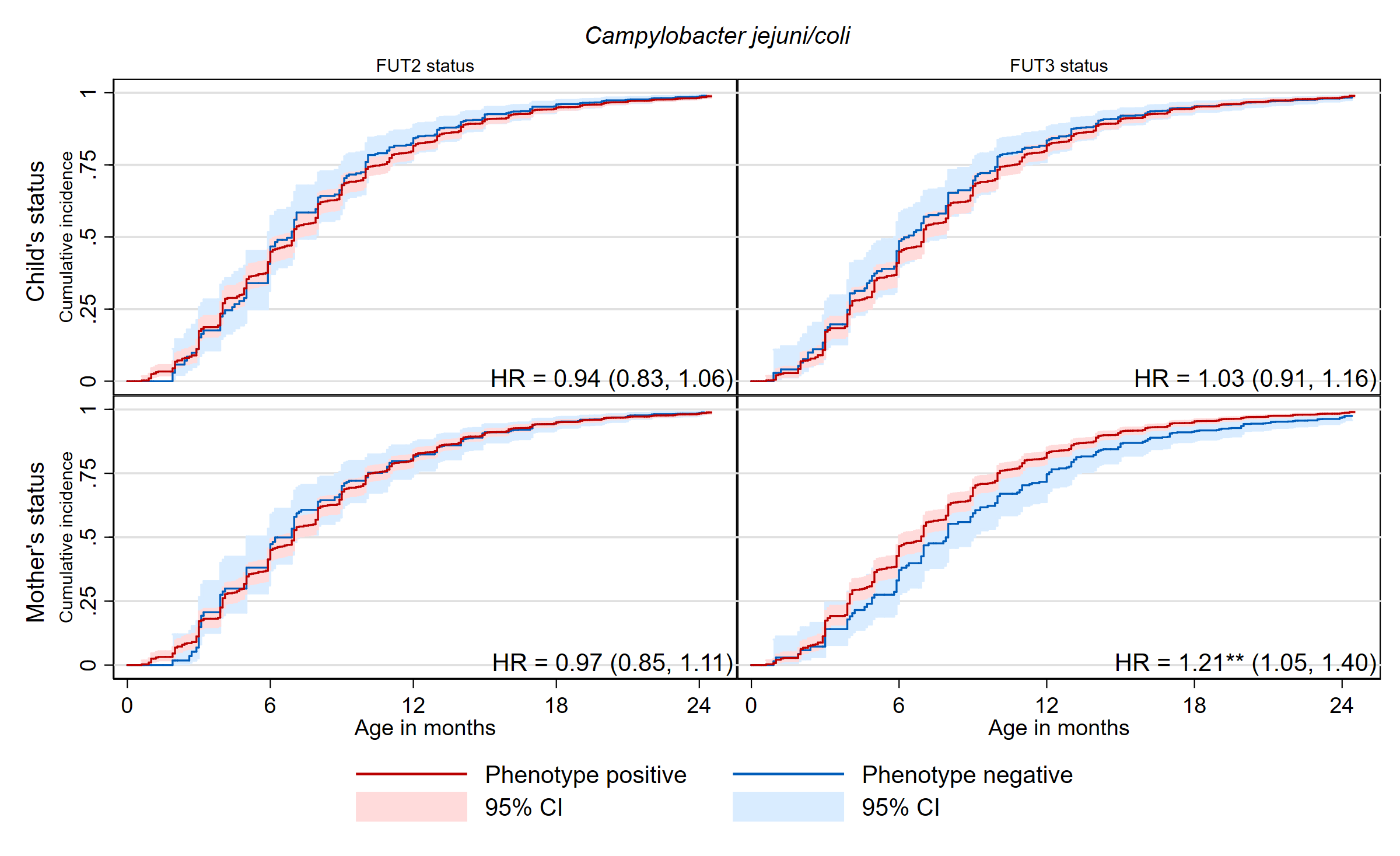


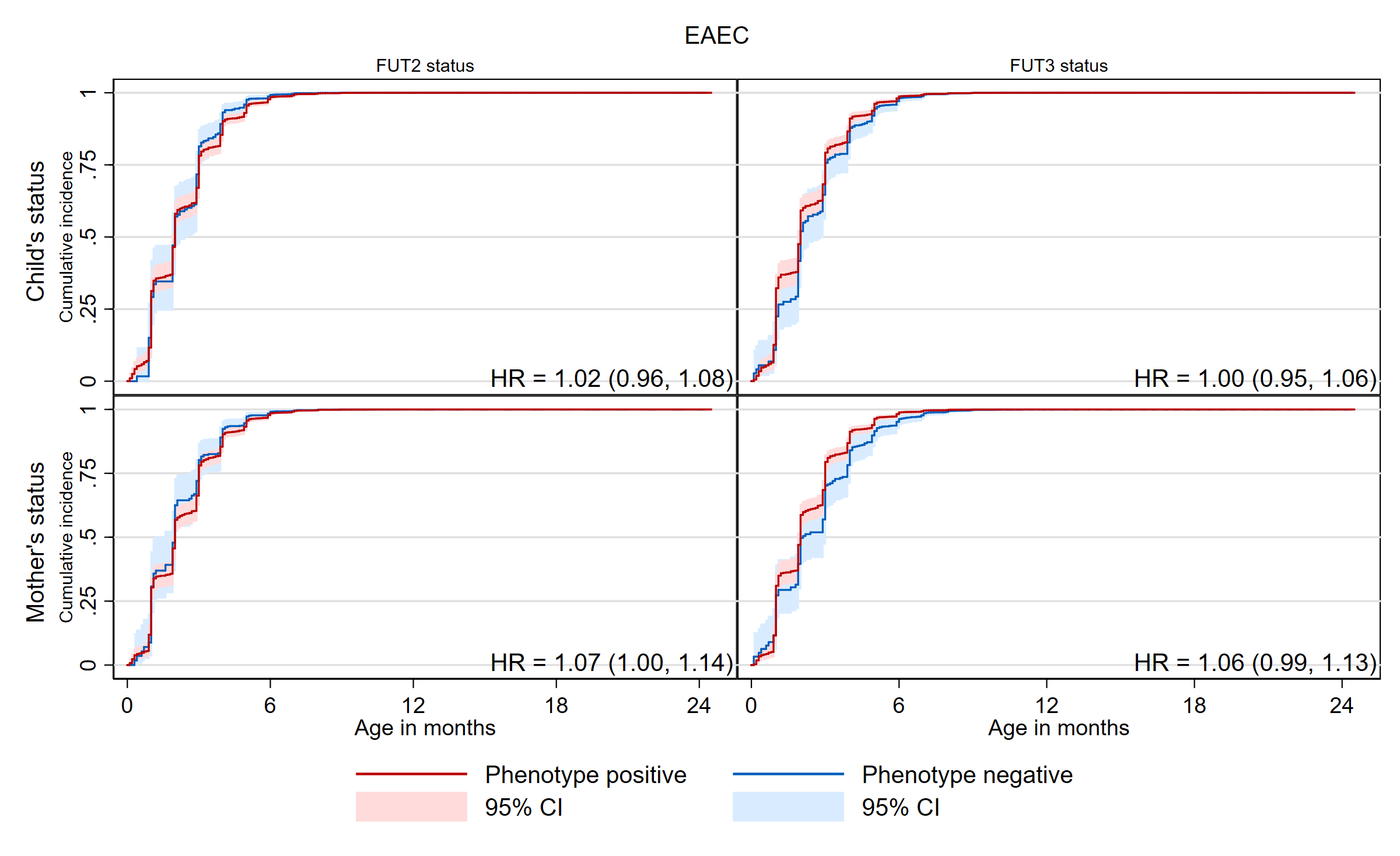


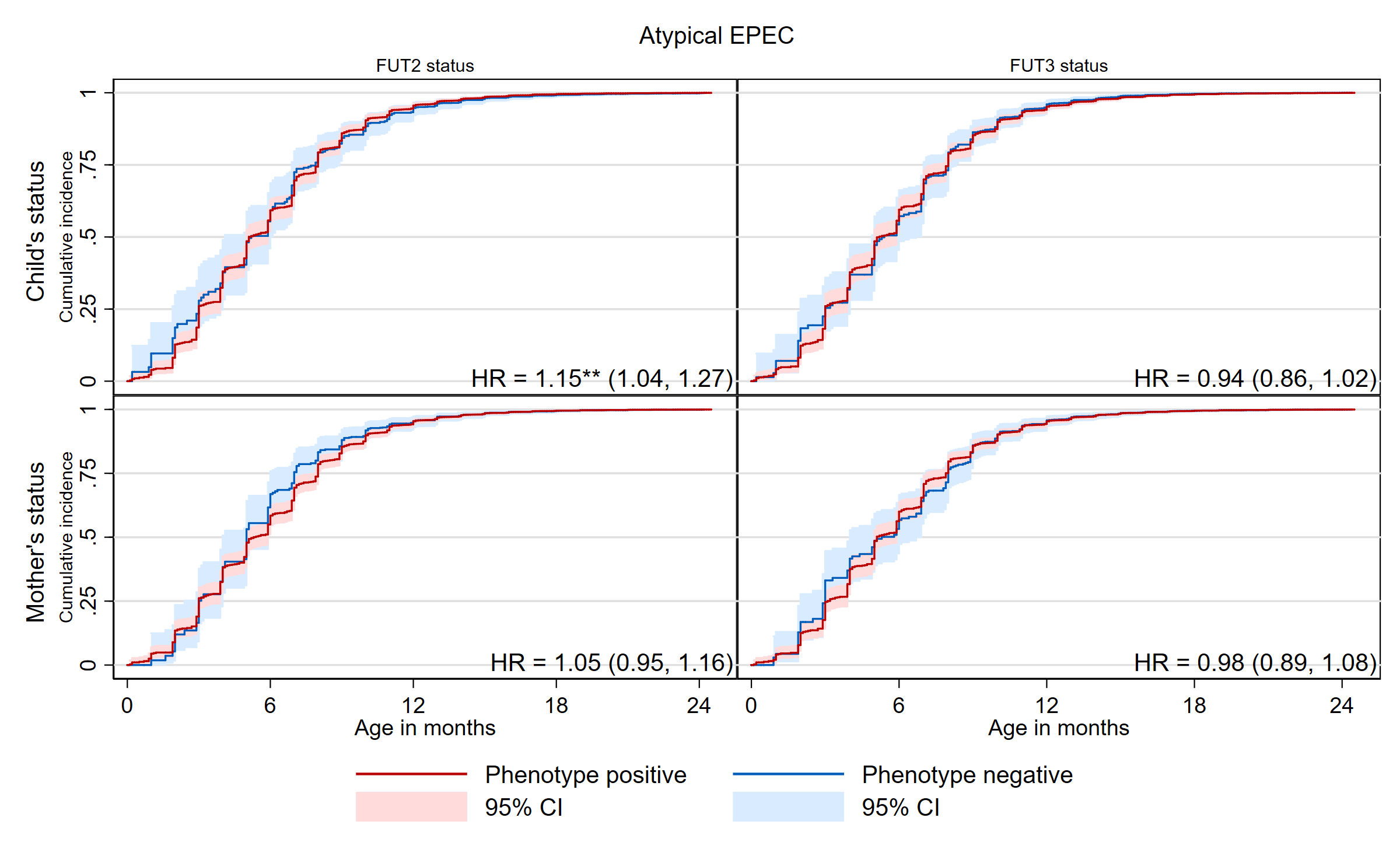


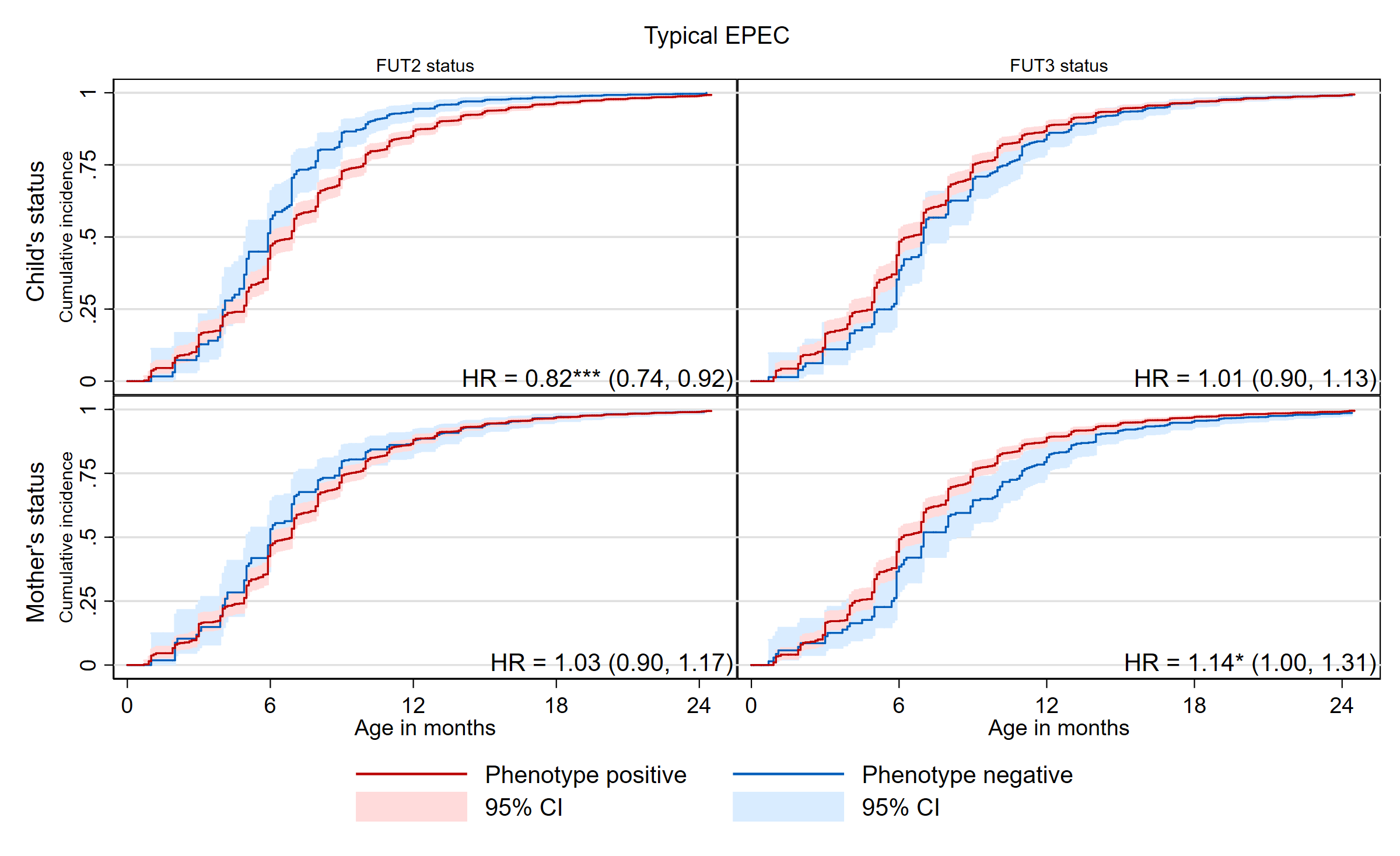


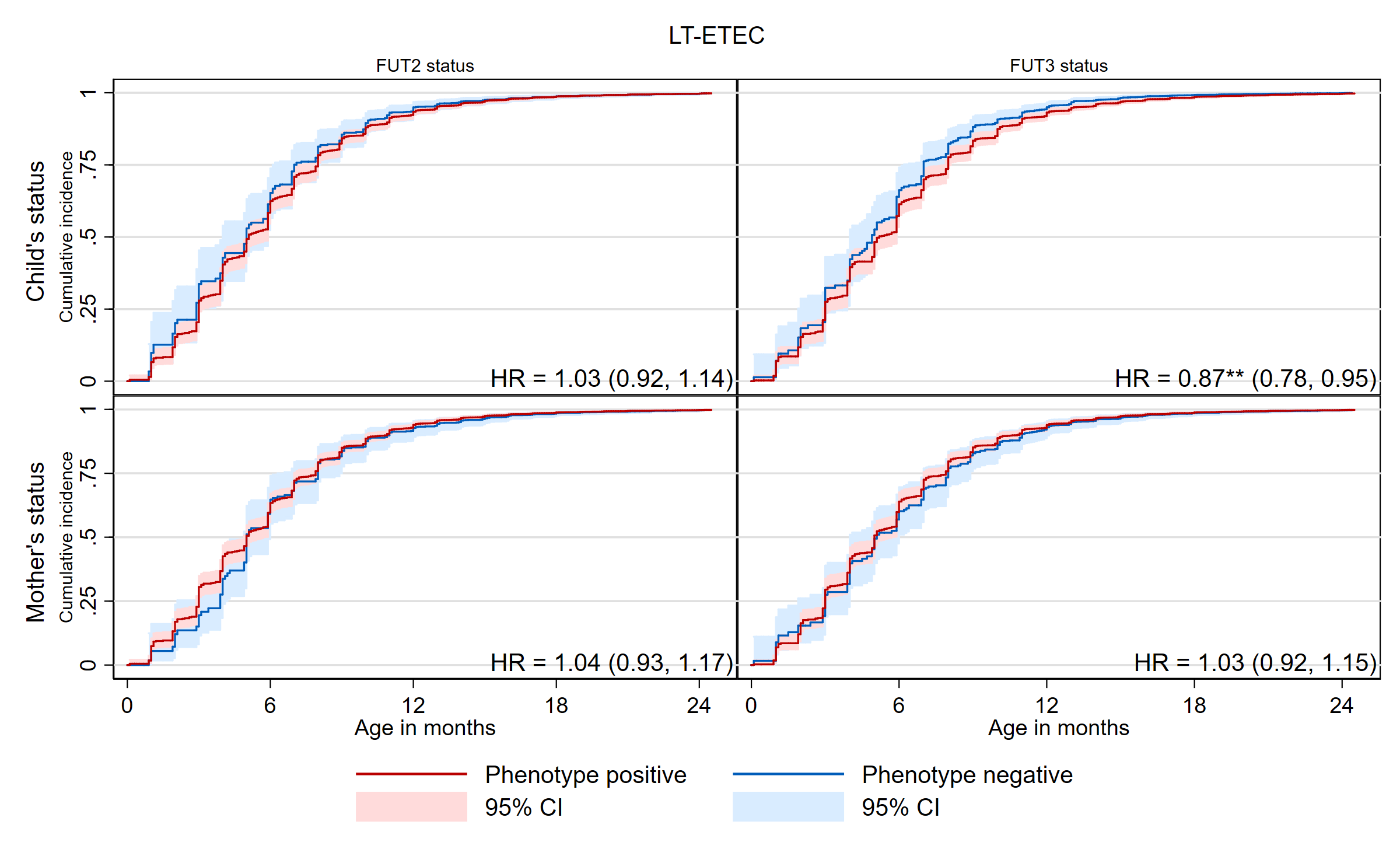


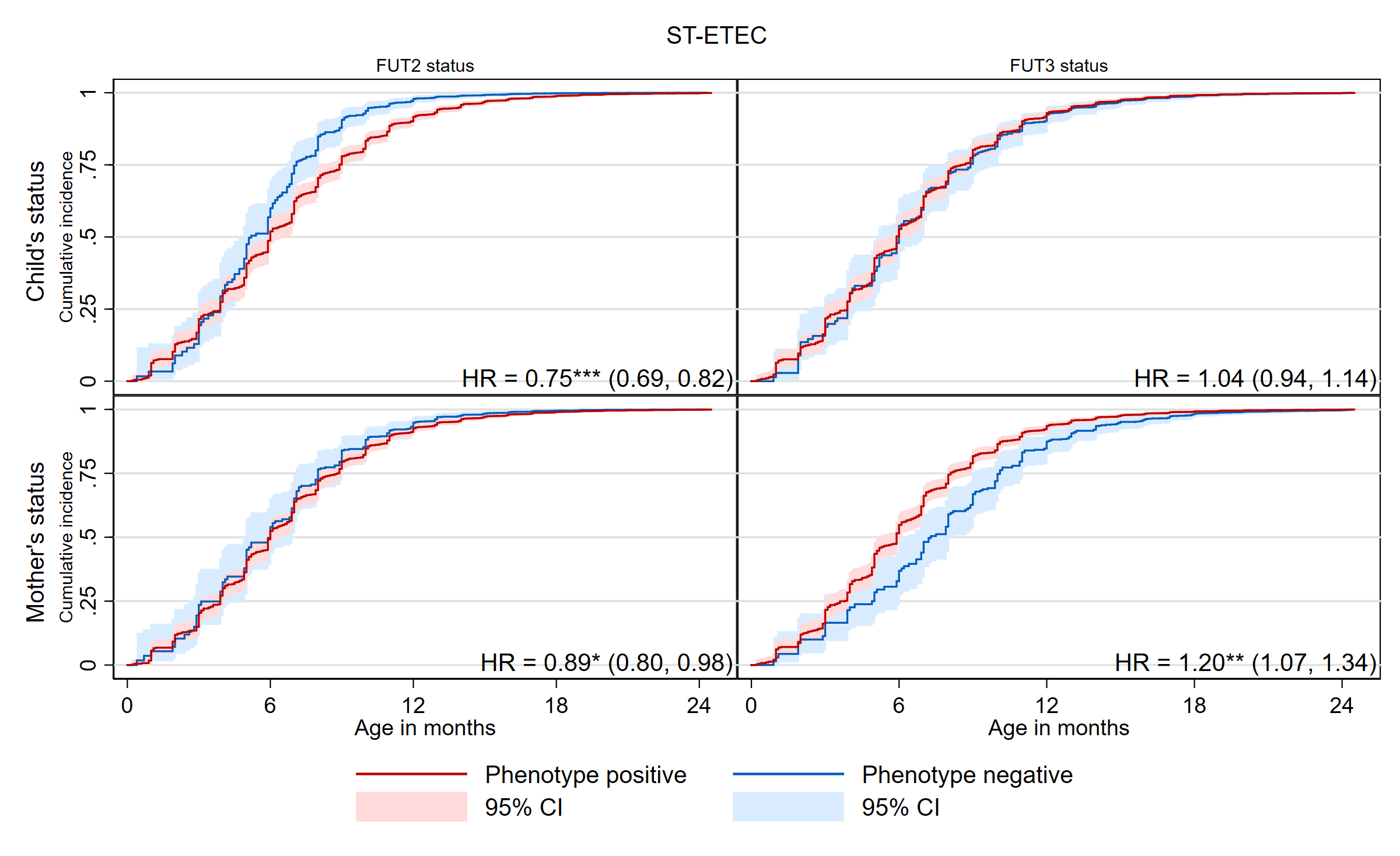


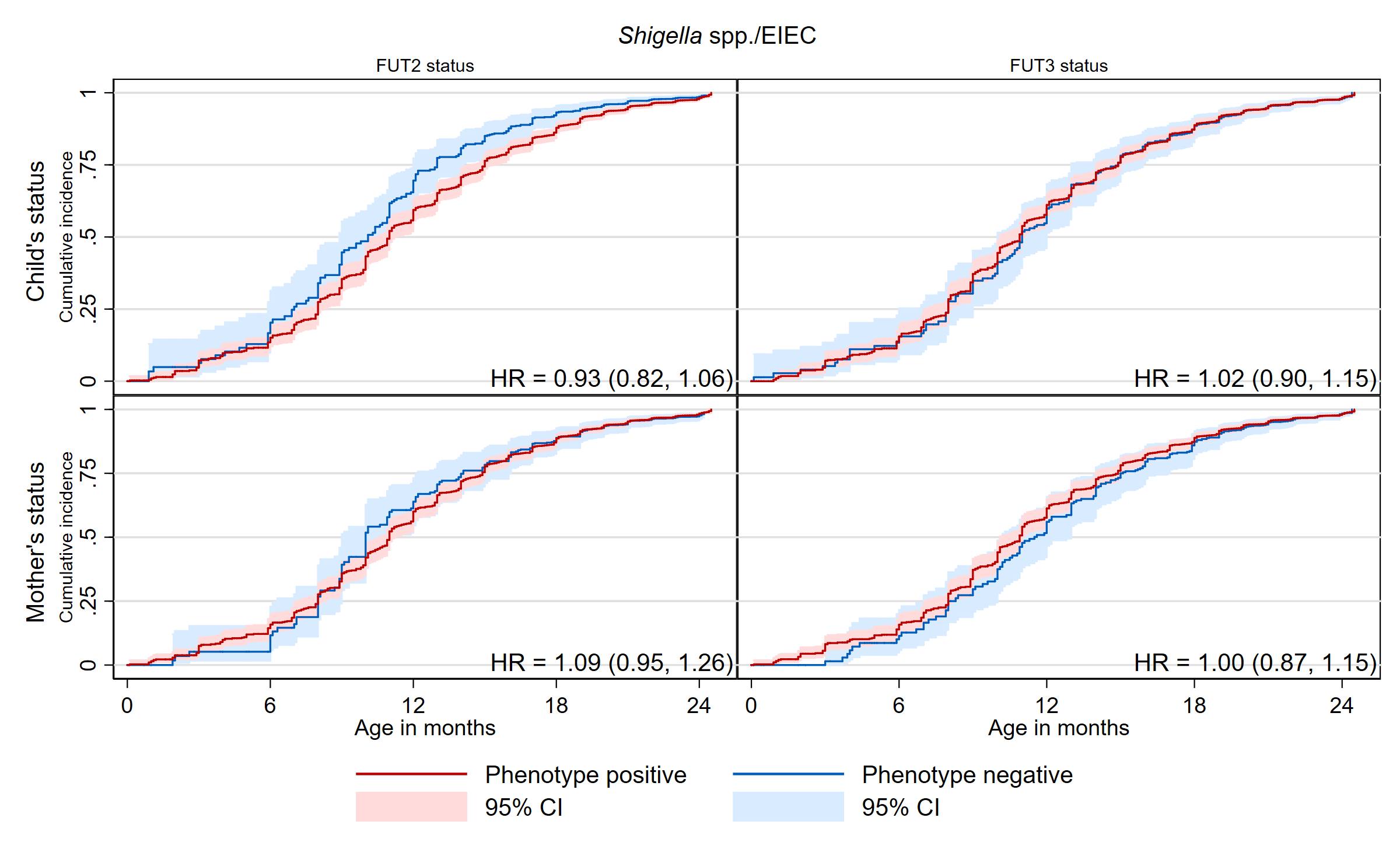


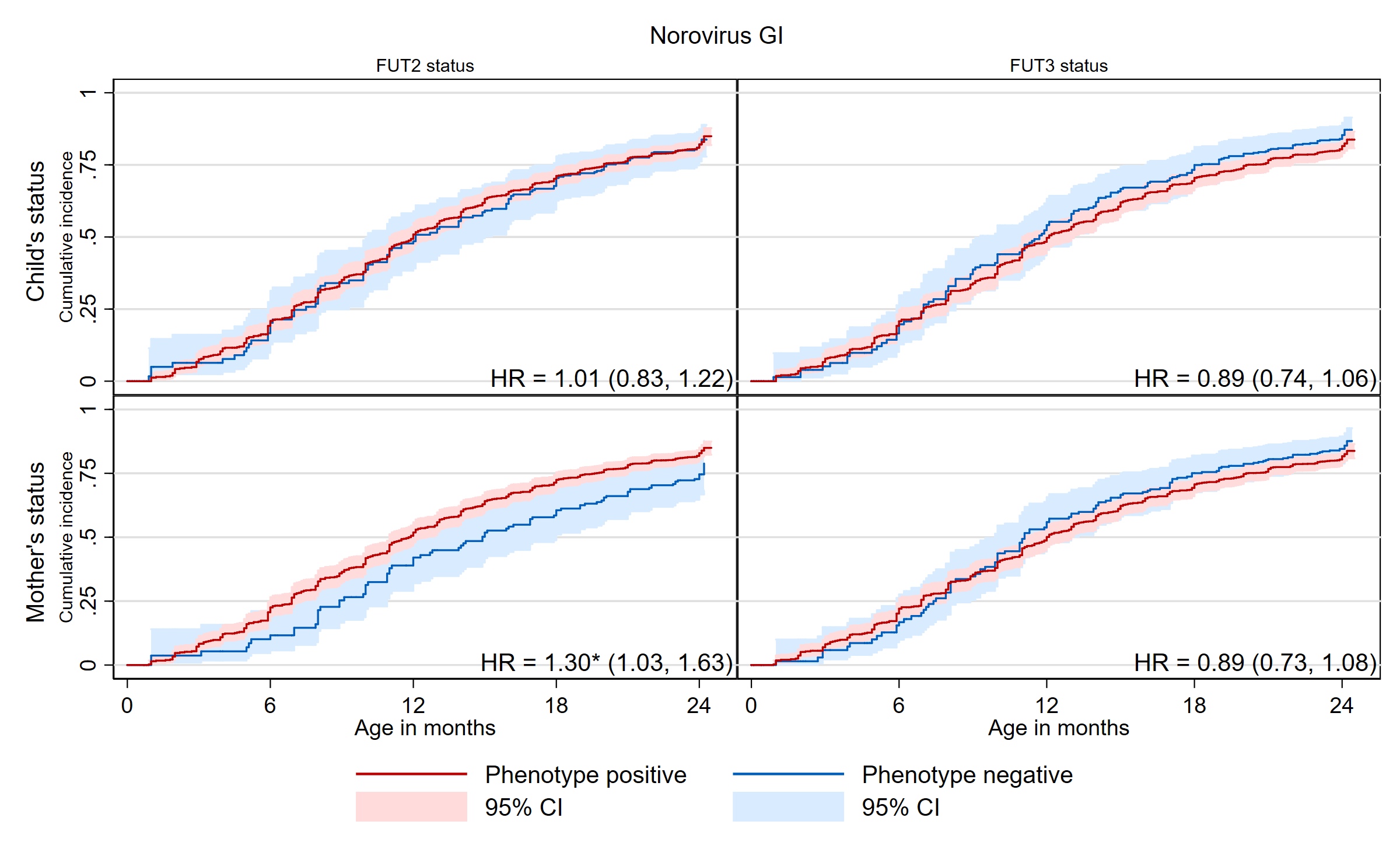


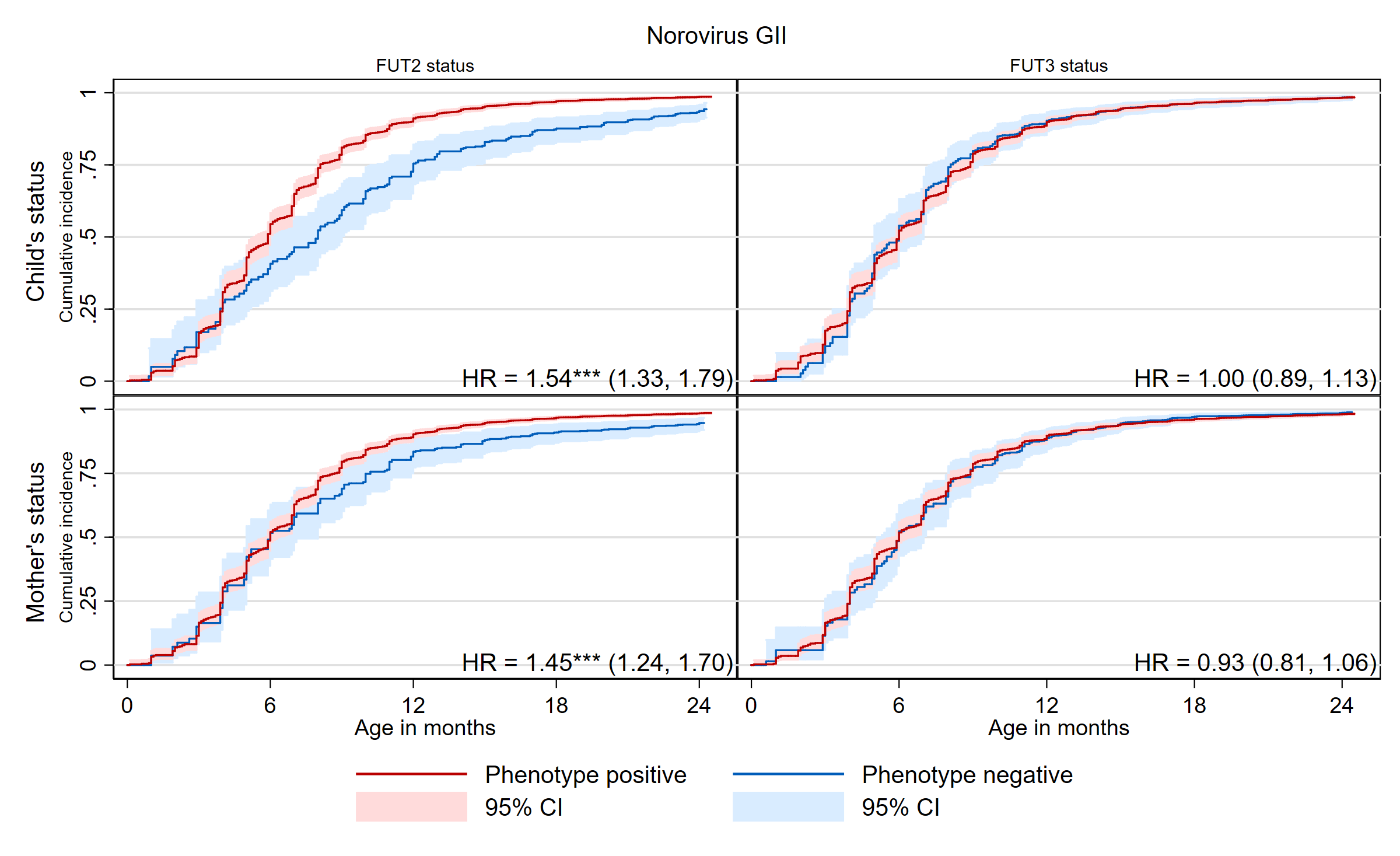


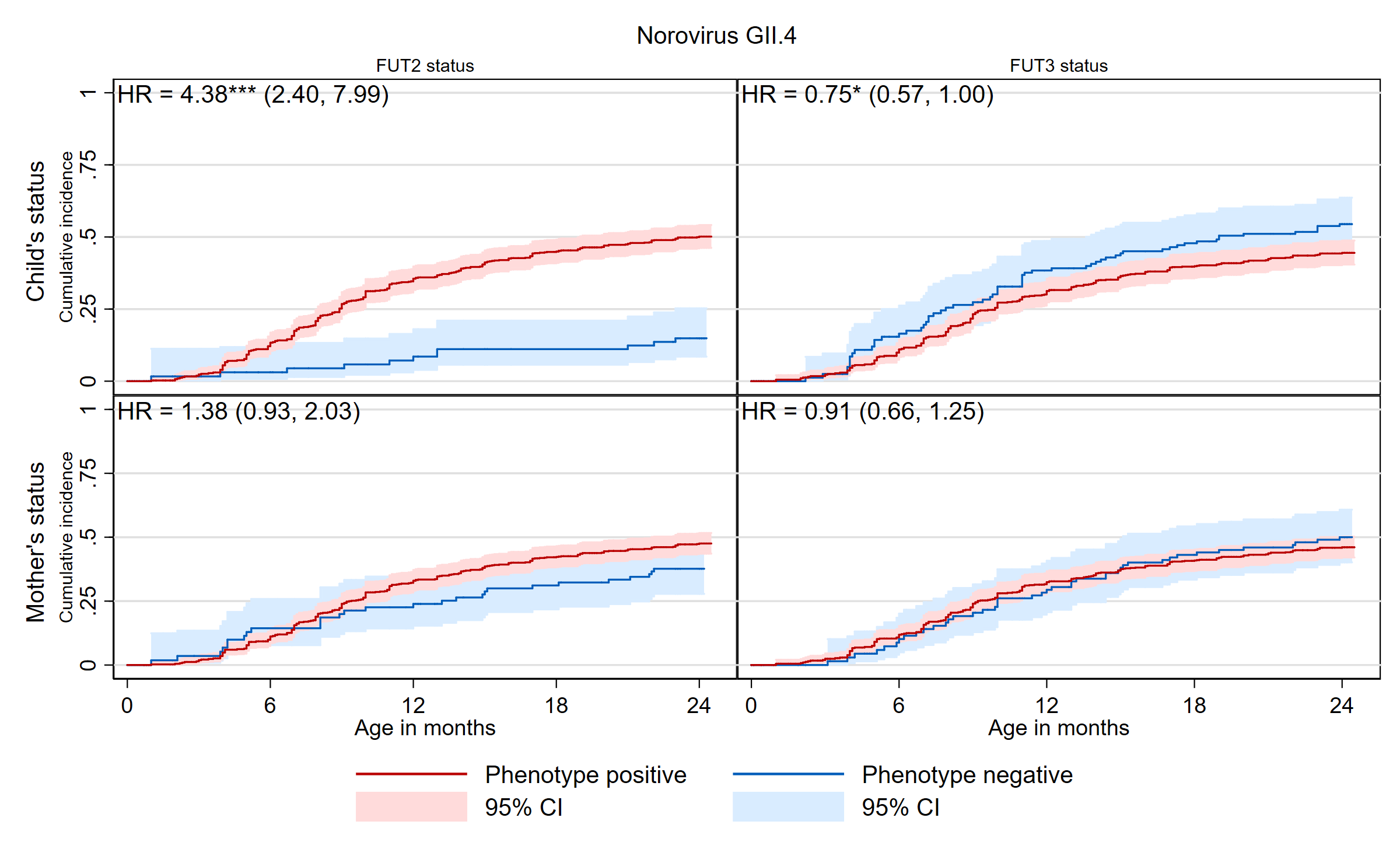


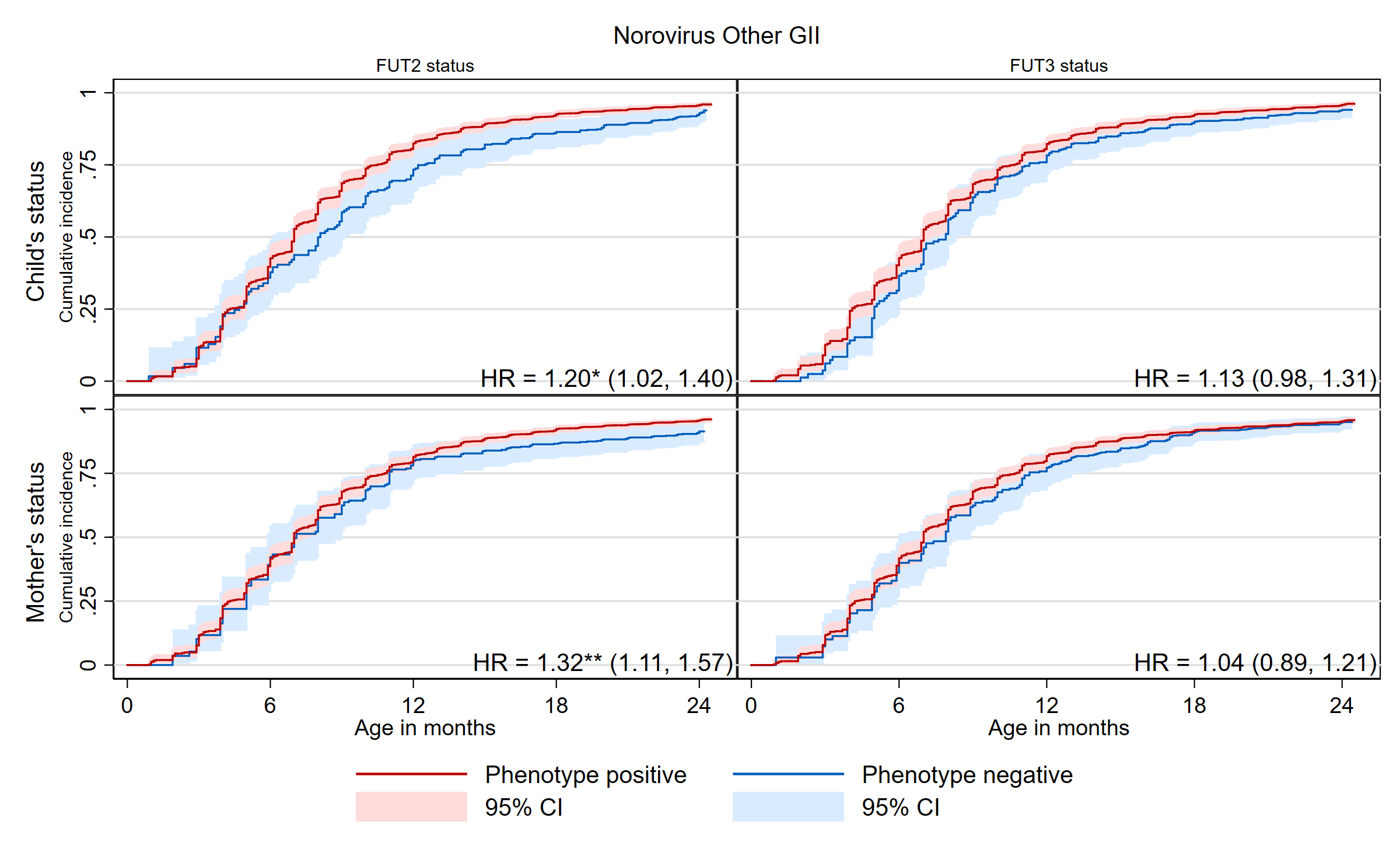


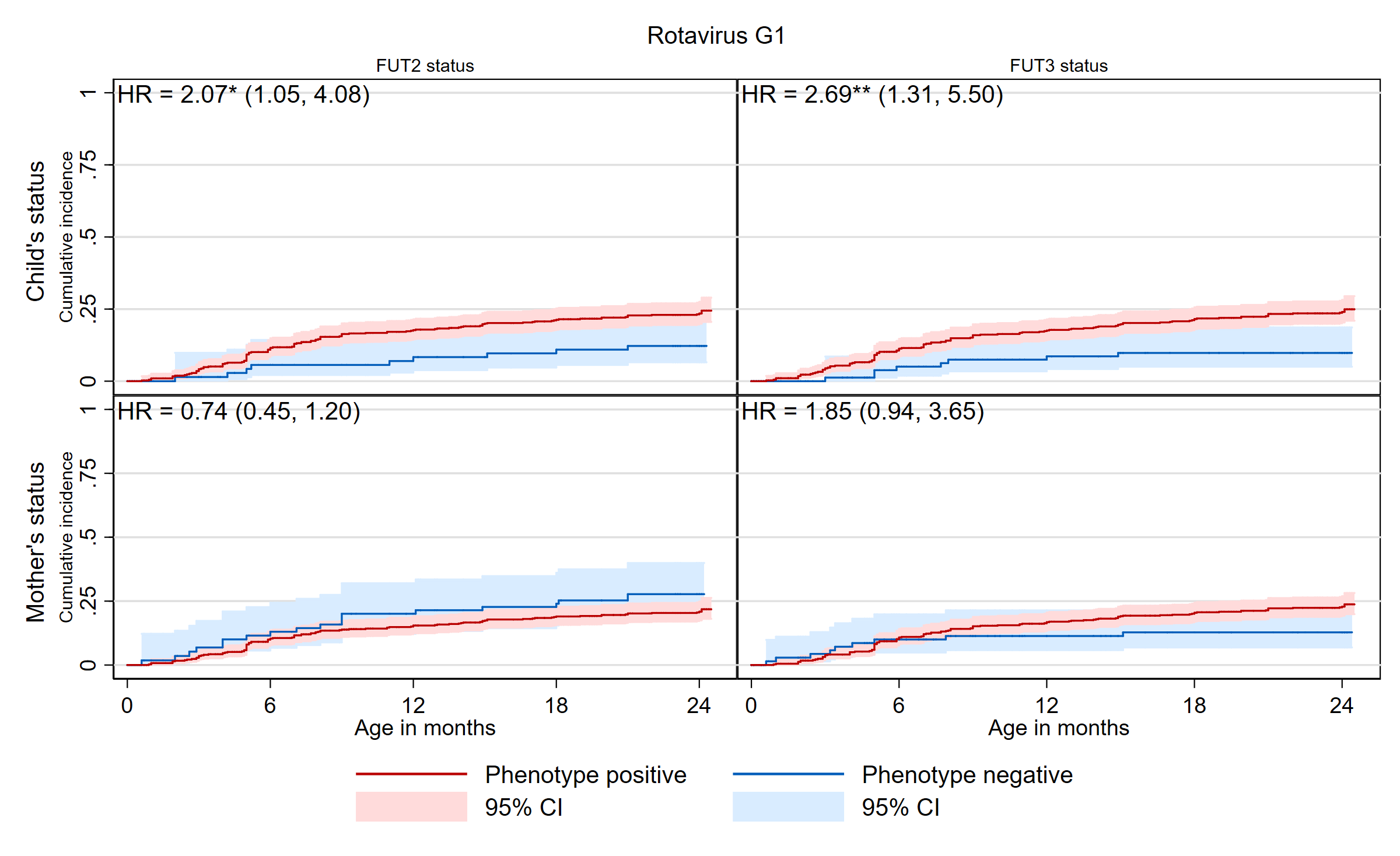


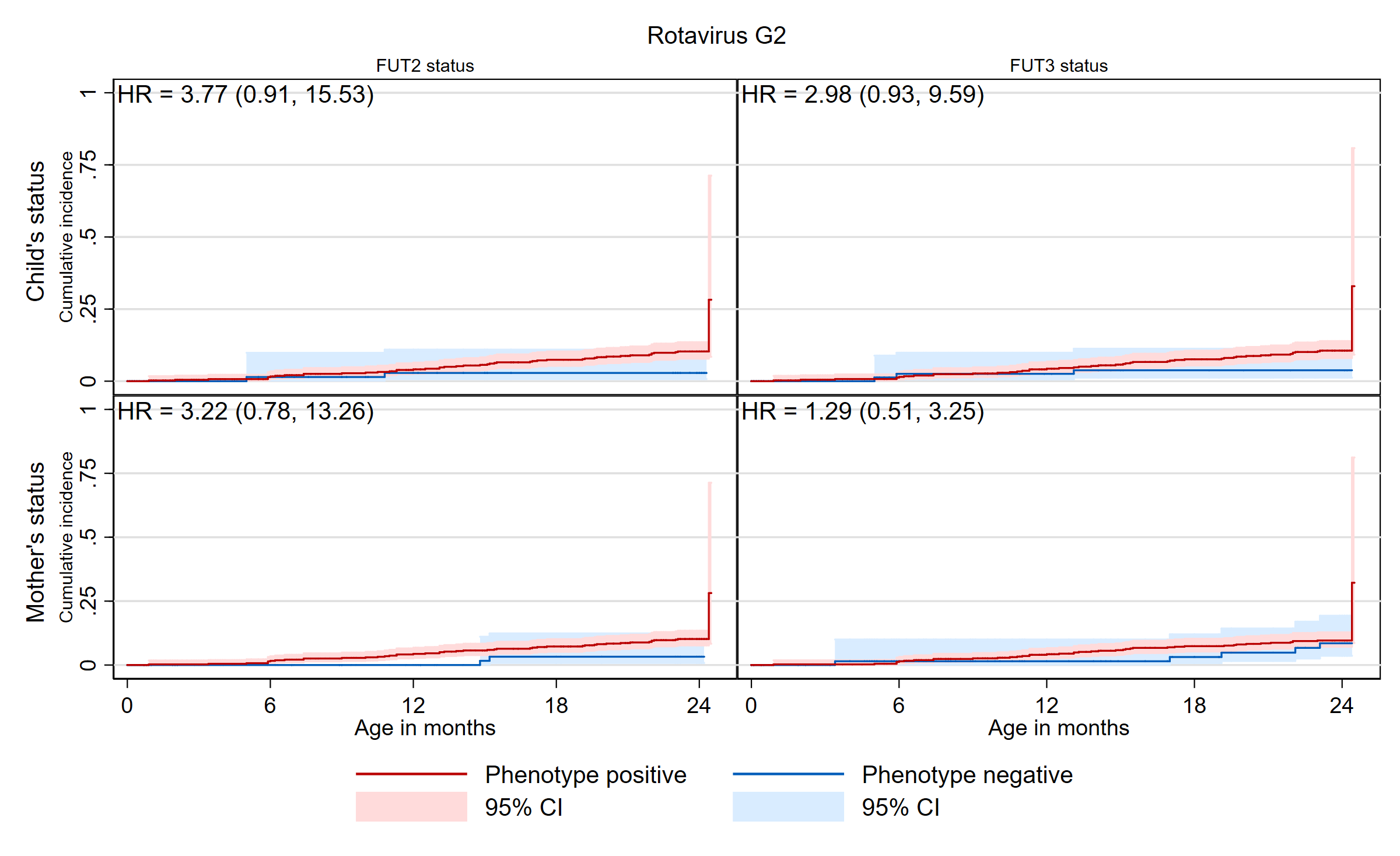


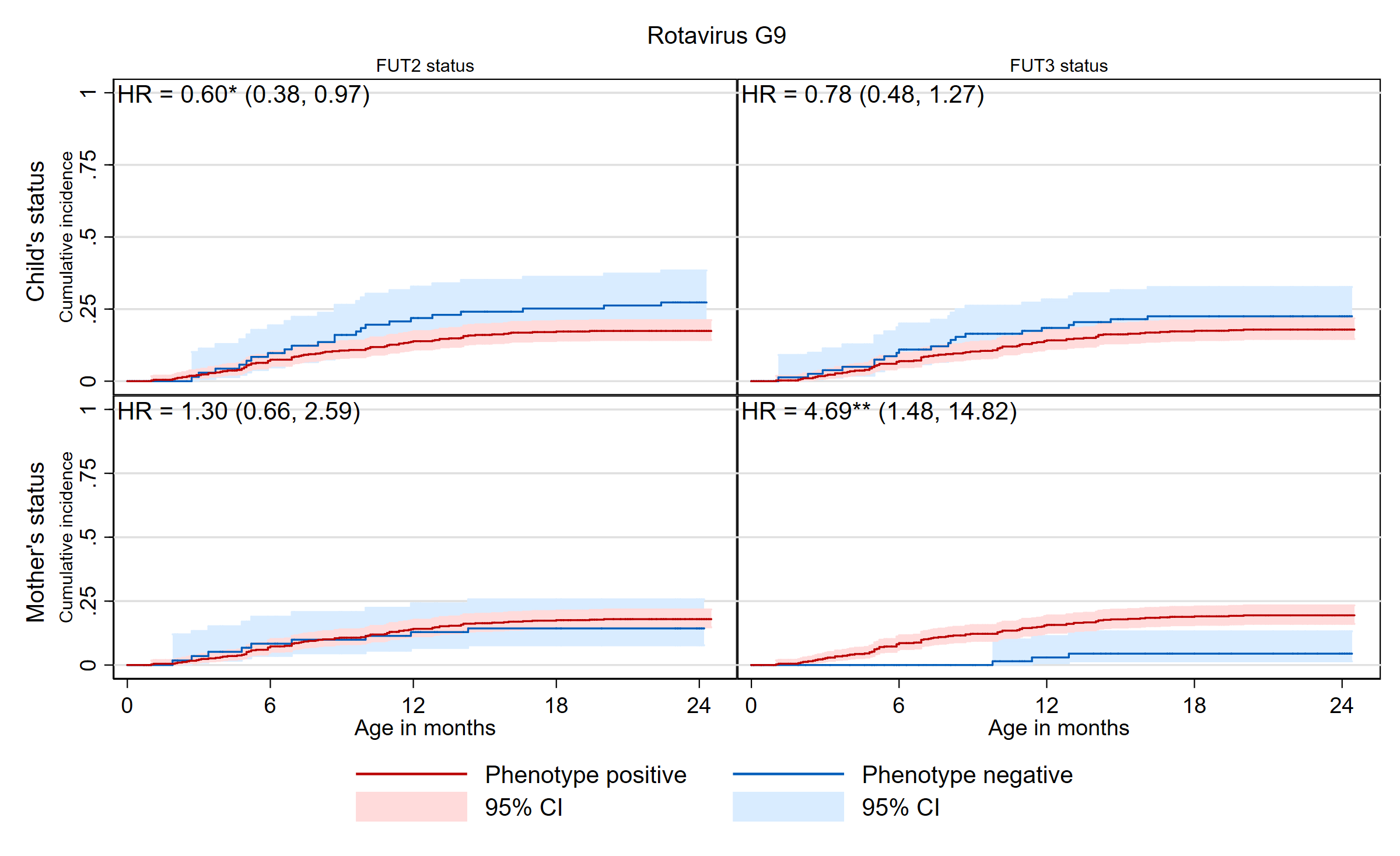


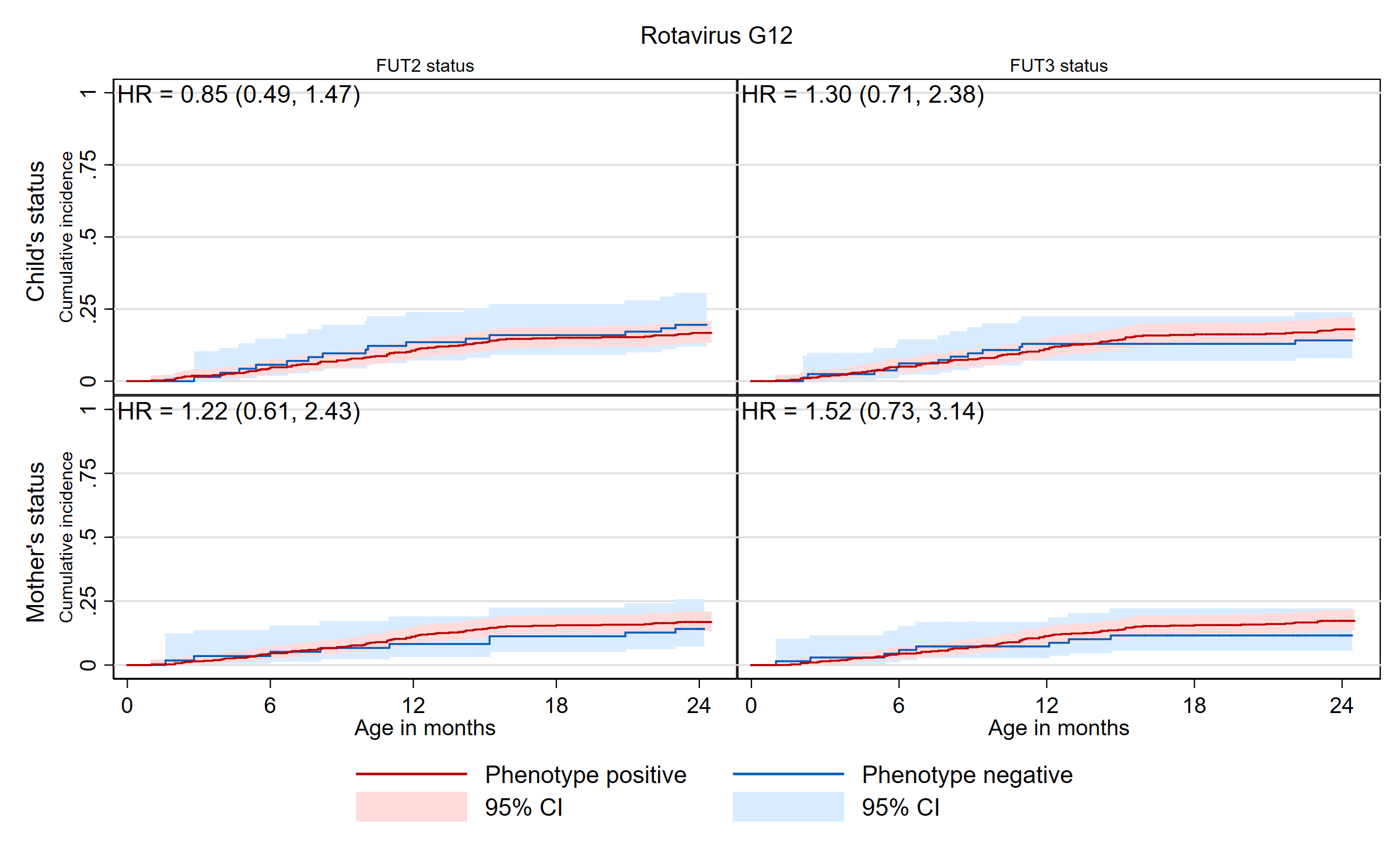


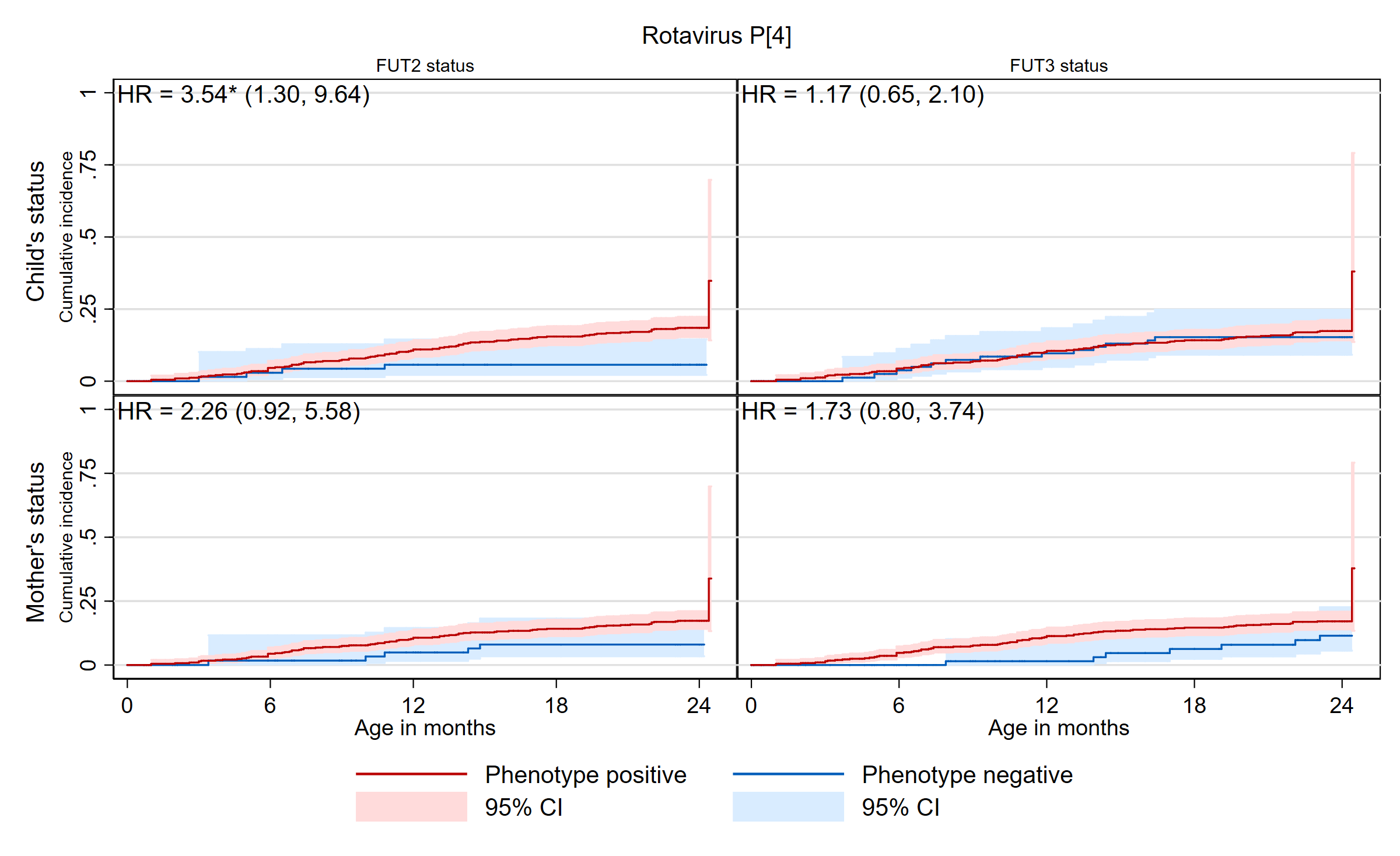


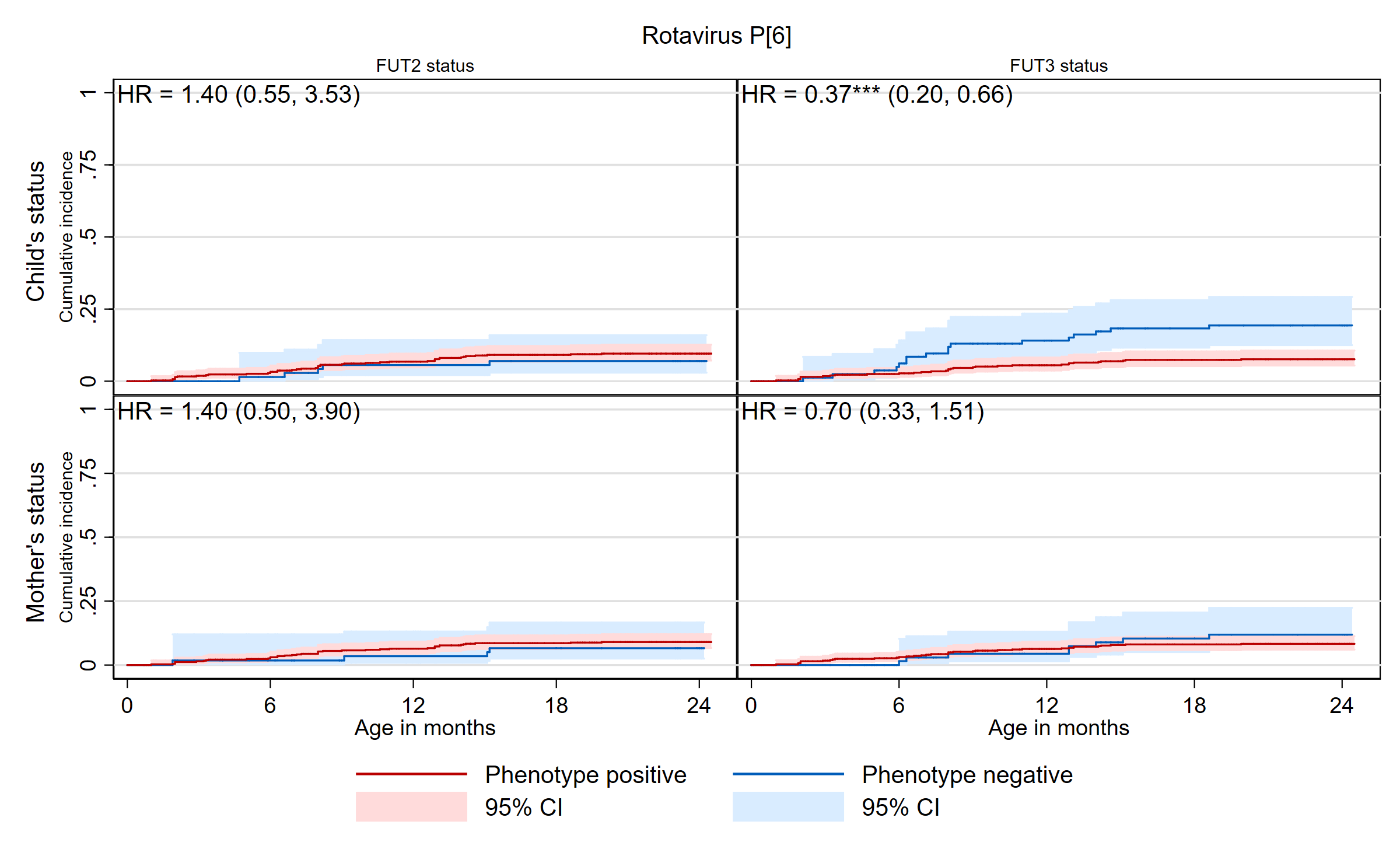


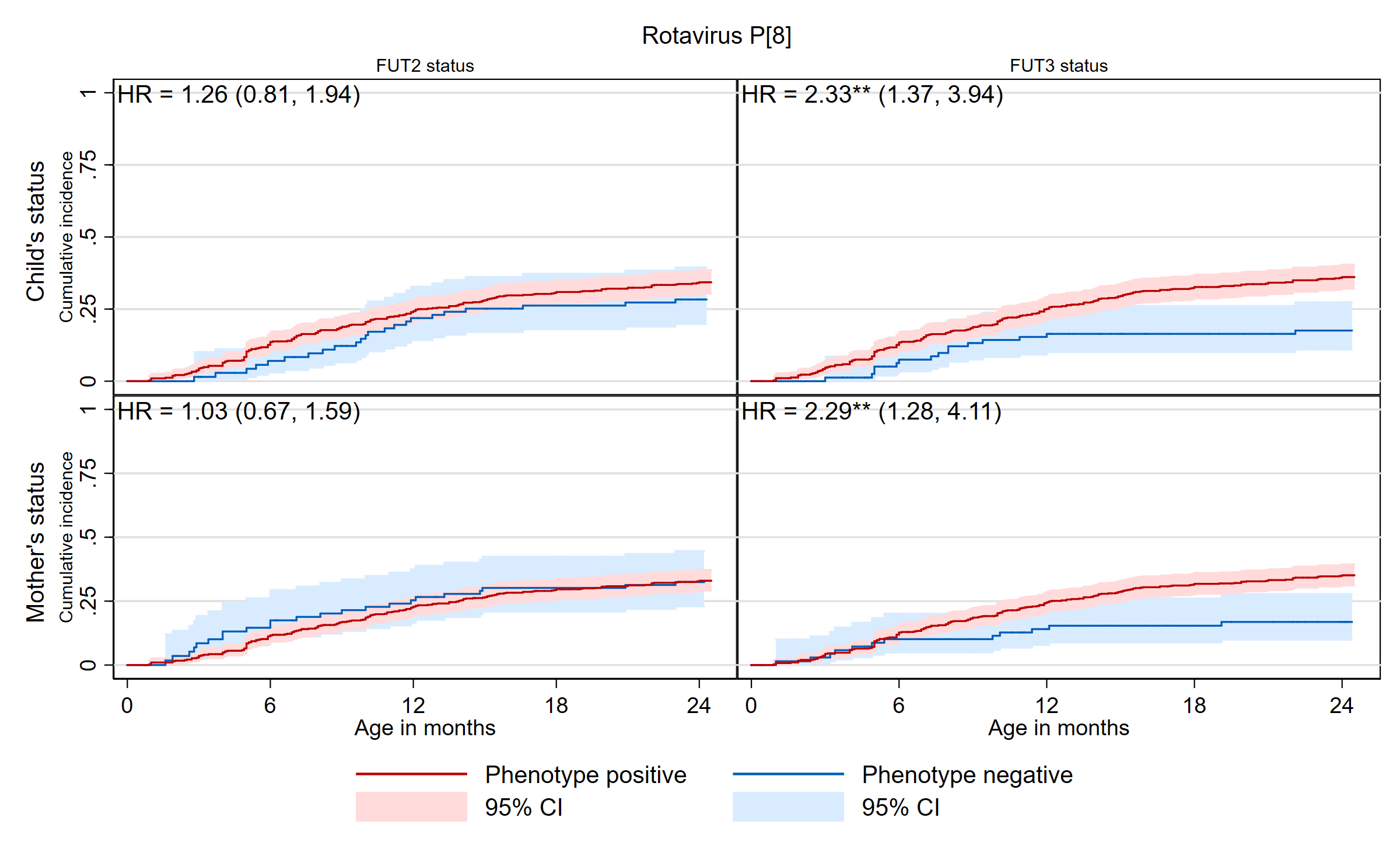


1. Bases in parentheses are heterozygous; * Base missing due to nested PCR approach (noisy) [↑](#footnote-ref-1)
2. Bases in parentheses are heterozygous; * Base missing due to nested PCR approach (noisy) [↑](#footnote-ref-2)
3. CI = Confidence Intervals, *** p<0.001, ** p=0.001 – 0.01, * p=0.01 – 0.05. [↑](#footnote-ref-3)
4. CI = Confidence Intervals, *** p<0.001, ** p=0.001 – 0.01, * p=0.01 – 0.05. [↑](#footnote-ref-4)
